# Si-Wu-Tang improves liver fibrosis by restoring liver sinusoidal endothelial cell functionality and reducing communication with hepatic stellate cells

**DOI:** 10.1186/s13020-024-01038-1

**Published:** 2024-12-31

**Authors:** Le Wang, Jiaorong Qu, Jianan Li, Xiaoyong Xue, Lingling Qin, Yufei Li, Yuanfeng Dou, Xiaohong Mu, Xiaojiaoyang Li

**Affiliations:** 1 School of Life Sciences, Beijing University of Chinese Medicine, 11 Bei San Huan Dong Lu, Beijing, 100029 China; 2 Department of Science and Technology, Beijing University of Chinese Medicine, 11 Bei San Huan Dong Lu, Beijing, 100029 China; 3 School of Chinese Materia Medica, Beijing University of Chinese Medicine, 11 Bei San Huan Dong Lu, Beijing, 100029 China; 4 Department of Orthopedics, Dongzhimen Hospital, Beijing University of Chinese Medicine, Beijing, China

**Keywords:** Si-Wu-Tang, Liver fibrosis, Liver sinusoidal endothelial cell, Angiogenesis, Cell adhesion, Defenestration

## Abstract

**Background:**

Liver fibrosis is a complex reparative process in response to chronic liver injuries, with limited effective therapeutic options available in clinical practice. During liver fibrosis, liver sinusoidal endothelial cells (LSECs) undergo phenotypic changes and also play a role in modulating cellular communications. Si-Wu-Tang (SWT), a traditional Chinese herbal remedy, has been extensively studied for its effectiveness in treating hematological, gynecological and hepatic diseases.

**Materials and methods:**

The component of SWT were identified by ultra-high-performance liquid chromatography (UHPLC). After establishing bile duct ligation (BDL)-induced liver fibrosis mice model and VEGFA-stimulated LSEC model, we invested the mechanism of SWT through RNA sequencing combined with molecular biology techniques.

**Results:**

SWT significantly improved the sinusoidal permeability and liver fibrosis induced by BDL and effectively regulated pathological processes in LSECs, such as angiogenesis, cell adhesion, basement membrane formation and defenestration. The anti-fibrosis effects of SWT were attributed to the inhibition on LSEC adhesion *via* COL8A1, on LSEC angiogenesis *via* IL-1β and the induction of LSEC defenestration by OLR1. Additionally, SWT disrupted the intercellular crosstalk between LSECs and hepatic stellate cells (HSCs) driven by IL-1β, thus alleviating liver fibrosis.

**Conclusion:**

SWT collectively ameliorated liver fibrosis by inhibiting the COL8A1/IL-1β/OLR1 pathways associated with LSEC angiogenesis, adhesion and defenestration, as well as suppressing LSEC secretion of IL-1β to reduce HSC activation.

**Supplementary Information:**

The online version contains supplementary material available at 10.1186/s13020-024-01038-1.

## Introduction

Liver fibrosis represents a dynamic reparative response of liver to various chronic injuries, characterized by an imbalance between collagen synthesis and metabolism within hepatic tissue, leading to excessive deposition of extracellular matrix (ECM). This buildup of ECM deposition causes structural alterations and functional impairment of the liver [6], making it a frequent target for therapeutic interventions in liver fibrosis [19]. Without timely treatment, liver fibrosis may progress to life-threatening complications such as cirrhosis and liver cancer, which results in approximately one million global deaths annually [30]. Hepatic stellate cells (HSCs) were usually considered as the culprit of liver fibrosis, which played an indispensable role in the process through their activation, proliferation, migration, and ECM deposition [12]. However, at present, there are a scarcity of clinically effective drugs targeting HSCs with satisfactory therapeutic outcomes. In recent years, multicellular communication and intrahepatic microenvironment regulation within the unique liver sinusoidal *Disse* space, where HSCs reside, have been increasingly recognized as potentially more significant role in liver fibrosis. Notably, as the creator of the *Disse* space, one of the non-parenchymal cells, liver sinusoidal endothelial cells (LSECs), not only independently regulate the advancement of liver fibrosis, but also act as gatekeepers for HSCs quiescence [8], which have garnered increasing attention from researchers.

We have recently elucidated the critical role of LSECs in liver fibrosis [26]. To be specific, during the initial phase of liver fibrosis, LSECs undergo dedifferentiation, marked by LSEC angiogenesis, strengthened intercellular connections and loss of fenestrations. Firstly, LSEC angiogenesis refers to the process in the liver where LSECs either form new vascular structures or facilitate the proliferation and regeneration of existing vasculature. The angiogenic factors such as vascular endothelial growth factor (VEGF), CD31, CD34, and von Willebrand factor (vWF) could promote pathological vascular formation of LSECs [34]. Secondly, cell adhesion in liver fibrosis affects disease progression by controlling cell-cell and cell-ECM interactions, further modulating tight junction formation between LSECs and basement membrane development. Collagen and laminin actively participate in the formation of the basement membrane and the process of intercellular adhesion. Adhesive molecules such as adhesive markers like fibronectin (FN1), Laminin beta-2 (LAMB2), coiled-coil domain-containing protein 80 (CCDC80) actively mediate adhesion processes [4, 10, 25]. Besides, during liver fibrosis, phenotypes of LSECs are also changed and characterized by a reduction or disappearance of fenestrae. As reported, plasmalemma vesicle-associated protein (PLVAP) is implicated in the formation of fenestration on the cell surface, regulating the exchange of blood and plasma components and influencing cellular permeability [14]. It is worth to mention that LSEC cytoskeleton serves a dual role in fenestrae formation. It not only maintains stability in cellular morphology but also directly influences the quantity and size of fenestrae, thus actively participating in the regulation of dynamic fenestrae changes. In addition, dedifferentiated LSECs also lose their ability to maintain HSC quiescence and instead activate HSCs *via* secreting various cytokines, growth factors and extracellular matrix molecules, thereby further promoting the progression of liver fibrosis. For instance, LSECs regulate the activation state and collagen synthesis of HSCs by releasing nitric oxide (NO) and endothelins. Also, inflammatory mediators such as Interleukin-1 and tumor necrosis factor alpha are also involved in the liver fibrosis process regulated by LSECs [2].

The limited efficacy of previous anti-fibrotic drugs may be partly attributed to their failure to comprehensive address the diverse fibrotic processes and regulate various cell types. Therefore, it is imperative to further explore therapeutic approaches that not only preserve normal cellular function but also disrupt intercellular communication, for a multidimensional improvement in the process of liver fibrosis. Si-Wu-Tang (SWT), a traditional classic formula comprising *Chuanxiong Rhizoma* (Chuanxiong), *Paeoniae Radix Alba* (Baishao), *Radix Angelicae Sinensis* (Danggui) and *Rehmanniae Radix Praeparata* (Shudihuang), originally utilized for treating gynecological disorders, has been extensively reported in recent years for its application in treating hepatobiliary diseases. A randomized, double-blind, placebo-controlled clinical trial have reported the antioxidant effects of SWT on the liver in healthy adults [7]. Our prior studies documented that SWT not only alleviated CCl_4_-induced liver injury by modulating gut microbiota and bile acid homeostasis but also improved bile duct ligation (BDL)-induced liver fibrosis by regulating the immune environment [22, 35]. Recently, we have discovered that SWT alleviated liver fibrosis through the H19-dependent pathways, thus modulating cytoskeleton remodeling and deposition of the ECM [27]. We also suggested that the therapeutic efficacy of SWT against BDL-induced liver fibrosis was mediated by various intrahepatic cell types, including HSCs, LSECs, and hepatocytes. However, it remained unclear whether SWT could targets LSECs and by what mechanisms. Additionally, apart from modulating fibrosis-related gene expression within LSECs and HSCs separately, the modulation of SWT in the intracellular communications between LSECs and HSCs is unknown.

This study aimed to explore the effects and mechanism of SWT on several bioprocesses of LSECs including LSEC angiogenesis, cell adhesion and fenestrae regulation, as well as the relation among these processes through RNA sequencing, bioinformatics analysis as well as in vivo, and in vitro experiments. In addition, we found SWT-controlled IL-1β expression was not only participated in the LSEC angiogenesis, but also involved in the cellular communication with HSCs. Our research might provide new insights into the pharmaceutical mechanism of SWT in the progress of liver fibrosis, by exploring the intricate involvement of LSECs related bioprocesses and LSECs-HSCs communication.

## Materials and methods

### Materials

The four herbs of SWT, including *Ligusticum chuanxiong* Hort. (Dry rhizome), *Paeonia lactiflora* Pall. (Dry root), *Angelica sinensis* (Oliv.) Diels (Dry root) and *Rehmannia glutinosa* Libosch. (Processed products of dry root tuber) were all purchased from Beijing Tongrentang (Group) Co. Ltd. (China) and identified by Prof. Liu from the School of Chinese Materia Medica, Beijing University of Chinese Medicine. Ursodeoxycholic acid (UDCA, U110695) was purchased from Aladdin (Shanghai, China). Recombinant rat VEGFA (CJ96) and Recombinant mouse/rat TGF-β1 (CK33) were purchased from Suzhou Novoprotein Technology Co., Ltd. (China). Recombinant Rat IL-1β (P6245) was purchased from Beyotime Biotechnology (Shanghai, China). Matrigel matrix (356234) was purchased from Corning Incorporated (New York, USA). TRITC Phalloidin (40734ES75) was obtained from Yeasen Biotechnology Co.,Ltd. (Shanghai, China). The antibodies used in the study were shown in Supplementary Table S1.

### SWT preparation and component identification

The four herbs of SWT in equal proportions were sliced and soaked in distilled water for 1 h, then decocted twice through the condensation reflux method. The liquid was concentrated using a rotary evaporator at 45 °C, then filtered twice through a 0.45 μm filter and stored at − 20 °C for later use. To further identify the active ingredients in SWT, we took a portion of the SWT liquid and diluted it to 0.064 g/ml with pure water, then extracted it with ultrasound for 1 h. Afterwards, the liquid was centrifuged at 4 °C, 12,000 rpm for 10 min to collect the supernatant, then it was filtered through a 0.22 μm filter. The centrifugation and filtration were repeated once more, and the filtrate was collected for subsequent analysis. We conducted mass spectrometric analysis of SWT by Ultra-high-performance liquid chromatography (UHPLC), which was performed in both positive and negative ion modes.

### Animal studies

All animal studies and procedures were approved by the Animal Ethics Committee of Beijing University of Chinese Medicine (BUCM-4-20200730023160). C57BL/6 J mice (9 weeks old, 22–25 g, male) were purchased from Beijing SIBEIFU Biotechnology Co., Ltd. China. The mice were bred under a regular light-dark cycle and provided with unlimited chow diet and sterile drinking water. The mice were randomly divided into six groups (n = 6): (1) sham group; (2) BDL group; (3) BDL + low-dose SWT group; (4) BDL + medium-dose SWT group; (5) BDL + high-dose SWT group; (6) BDL + UDCA group. In the group (1), mice underwent sham surgery, while in the group (2)-(6), mice underwent BDL surgery. The specific modeling and dosing schemes refer to previous studies [27, 35]. The low (L), medium (M), high (H) doses of SWT and the dose of UDCA were 2.6 g/kg, 5.2 g/kg, 10.4 g/kg and 40 mg/kg, respectively. In groups (3)-(6), mice were pre-treated with SWT or UDCA (i.g.) for 3 days before undergoing the BDL surgery. There was no intervention during the first 3 days after surgery. From day 4 to day 7 of post-surgery, mice were continuously treated with SWT or UDCA (i.g.) for 4 days. After the final treatment, 24 h later, the mice were anesthetized and euthanized. Blood and liver samples were collected for subsequent research.

### RNA-sequencing and bioinformatics analysis

Partial mouse liver tissue (30 mg) was used for total RNA extraction, quantified using the NanoRhatometer@ spectrophotometer (IMPLEN, USA). Then mRNA was purified and cDNA fragments of 250–300 bp were synthesized. As previously reported, sequencing library was produced on the Illumina Novaseq platform [16]. Gene expression data standardization and differentially expressed genes were identified through the edgeR package. Differential expression genes classification, heatmaps and Gene Ontology (GO) enrichment analysis was performed using R software.

### Cell culture

The cell line of rat LSEC (BNBIO, Beijing, China) was cultured in DMEM containing 10% fetal bovine serum (Corning) and 1% penicillin-streptomycin (P-S). Rat hepatic stellate cell line HSC-T6 (BNBIO, Beijing, China) was cultured in DMEM containing 10% fetal bovine serum (Bioind) and 1% P-S. All cells used for in vitro experiments are within the 8th passage. Based on the previous study [36] and our experimental results, we utilized VEGFA (60 ng/ml) to establish in vitro fibrotic model of LSECs. Referring to our previous study [23], we determined the concentrations of SWT for the in vitro experiments as 25 µg/ml (L), 50 µg/ml (M), and 75 µg/ml (H). For the conditioned medium co-culture experiment, LSECs were cultured in six-well plates for 24 h, treated with drugs for 24 h, and then the conditioned medium from LSECs were collected for further culture of HSCs in six-well plates. After 24 h, HSCs were collected for subsequent qPCR and immunofluorescence experiments.

### Cell viability assay

LSECs were seeded at a concentration of 10^4^ cells per well in a 96-well plate. Once the cell confluence reached approximately 70%, varying concentrations of SWT were applied for a duration of 24 h. Cell viability was assessed using the cell counting kit-8 (CCK8) (LABLEAD, Beijing, China). The culture medium in the 96-well plate was replaced with 100 µl of the CCK8 working solution per well and incubated for 1 h at 37 °C. The optical density (OD) was measured at a wavelength of 450 nm using a microplate reader.

### Colony formation assay

The pre-treated LSECs were digested and resuspended, then seeded at a density of 700 cells per well in a six-well plate. The LSECs were continuously cultured for 14 days. During the culture period, the medium was replaced every three days, and the cell status was observed. Upon completion of the culture, the LSECs were fixed with 4% paraformaldehyde for 15 min, followed by staining with 0.1% crystal violet solution for an additional 15 min. The LSECs were then gently washed with PBS to remove excess staining solution. Finally, observations and recordings were taken under the microscope.

### Immunohistochemistry and immunofluorescence staining

Mouse liver tissue sections were dewaxed, hydrated, and antigen retrieval was performed through EDTA antigen retrieval solution. Endogenous peroxidase activity was quenched *via* 3% H_2_O_2_, tissue sections were then incubated overnight at 4 °C with a primary antibody against Fibronectin (diluted 1:200). The next day, tissue sections were incubated with goat anti-mouse/rabbit IgG HRP polymer secondary antibody (ZSGB-BIO, Beijing, China). After DAB staining and counterstaining with hematoxylin, sections were dehydrated, mounted with neutral gum, and imaged though the A009 super-resolution microscopy tissue imaging system (Leica Aperio Versa). The specific experimental procedures of immunofluorescence are referenced in our previous article [16], including animal liver tissue slicing and cell immunofluorescence. Images were acquired using the Olympus FV3000 confocal laser scanning microscope (Tokyo, Japan) after completion of the experiments.

### Tube formation assay

Matrigel matrix was covered onto pre-chilled 96-well plates with 50 µl per well (200 µg/ml), and then incubated at 37 °C for 1 h to allow gelation. LSECs were added to the 96-well plate at a density of 1 × 10^4^ per well and cultured for 24 h in a cell culture incubator. Subsequently, the culture medium was replaced with treat medium containing 1% serum and stabilized for 1 h. After being treated with different reagents sequentially, the plate was returned to the cell culture incubator for another 24 h. Finally, after replacing the treat medium with PBS solution, the tubular formation of LSECs was observed under a microscope and recorded with image J.

### Cell adhesion experiment

The 96-well plate was first coated with 10 µg/ml collagen at room temperature for 1 h, after that, it was recoated with 1% heat-denatured BSA (200 µl/well) and incubated at 37 °C for 1 h. Afterward, the plate was washed twice with DMEM culture medium, and subsequently, LSECs treated with various agents were digested, resuspended, and seeded into the 96-well plate (5 × 10^4^ per well), followed by incubation at 37 °C for 1 h. After the non-adherent cells were washed away with PBS solution, the cell count was detected and the adhesion rate was calculated using the CCK8. The OD values determined by the CCK8 method were directly proportional to the number of adherent cells.

### Detection of NO

The fundamental principle is to indirectly determine the concentration of NO by measuring the amount of nitrite (NaNO2) produced through the oxidation of NO in the solution. Mouse liver tissue and LSEC samples were rapidly lysed on ice using cell and tissue lysis buffer (Beyotime, Shanghai, China), then centrifugated at 10,000 g for 5 min to collect the supernatant for NO detection. The supernatant from cell culture medium was directly used for NO content measurement. Experimental procedures were performed according to the instructions of the NO assay kit (Beyotime, Shanghai, China).

### IL-1β content detection

The cell culture supernatant was centrifuged at 1000 g for 10 min to remove particles and aggregates, retaining the upper liquid for IL-1β content detection. Experimental procedures were conducted following the instructions of the rat IL-1β ELISA kit (Biorigin, Beijing, China).

### Statistical analysis

All experimental data were repeated at least three times and processed and analyzed *via* GraphPad Prism 8 software. The results were presented as mean ± SEM, and differences between groups were analyzed by one-way analysis of variance (ANOVA). A *P*-value less than 0.05 was considered statistically significant. Other methods were shown in the supplementary document.

## Results

### The identification of the representative components of SWT

**Fig. 1:**
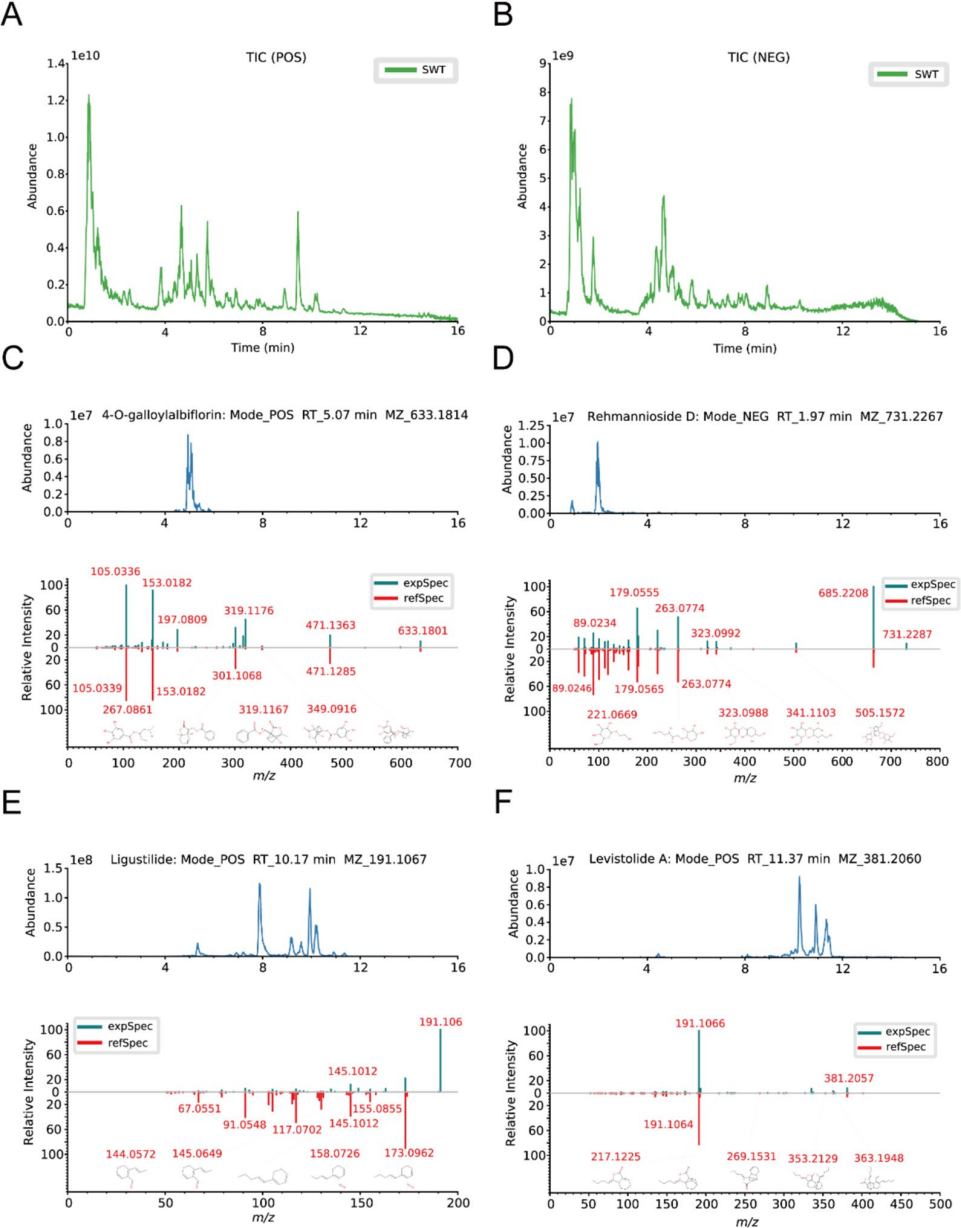
The total ion chromatography (TIC) of SWT and the mass spectra of representative compounds. **A**,** B** TIC of SWT in positive ion mode (**A**) and negative ion mode (**B**). **C–F** Mass spectrum of 4-O-galloylalbiflorin (**C**), rehmannioside D (**D**), ligustilide (**E**) and levistolide A (**F**)

Firstly, we employed UHPLC technology coupled with quadrupole-orbitrap high-resolution mass spectrometry to evaluate the quality of SWT we prepared. The total ion chromatograms in both positive and negative ion modes were depicted in Fig. 1A and B. Simultaneously, we selected four major components, namely 4-O-galloylalbiflorin, Rehmannioside D, Ligustilide and Levistolide A as representatives of SWT, and analyzed their quasi-molecular ions and fragment ions. Through data analysis, the representative component of *Paeoniae Radix Alba*, 4-O-galloylalbiflorin was identified by a quasi-molecular ion peak at m/z 633.1814 with a retention time of 5.07 min (Fig. 1C). Rehmannioside D, the representative component of *Rehmanniae Radix Praeparata* showed a quasi-molecular ion peak at m/z 731.2267 in the negative mode (Fig. 1D). Besides, the representative component of *Chuanxiong Rhizoma*, ligustilide exhibited a quasi-molecular ion at m/z of 191.1067 with a retention time of 10.17 min (Fig. 1E). And a quasi-molecular ion with m/z of 381.2060 and retention time of 11.37 min was identified as levistolide A, the active component of *Angelica Sinensis* (Fig. 1F). The main components of SWT, as identified, were shown in Supplementary table S2.

### SWT significantly alleviates BDL-induced liver fibrosis in mice and improves hepatic sinusoidal permeability

**Fig. 2:**
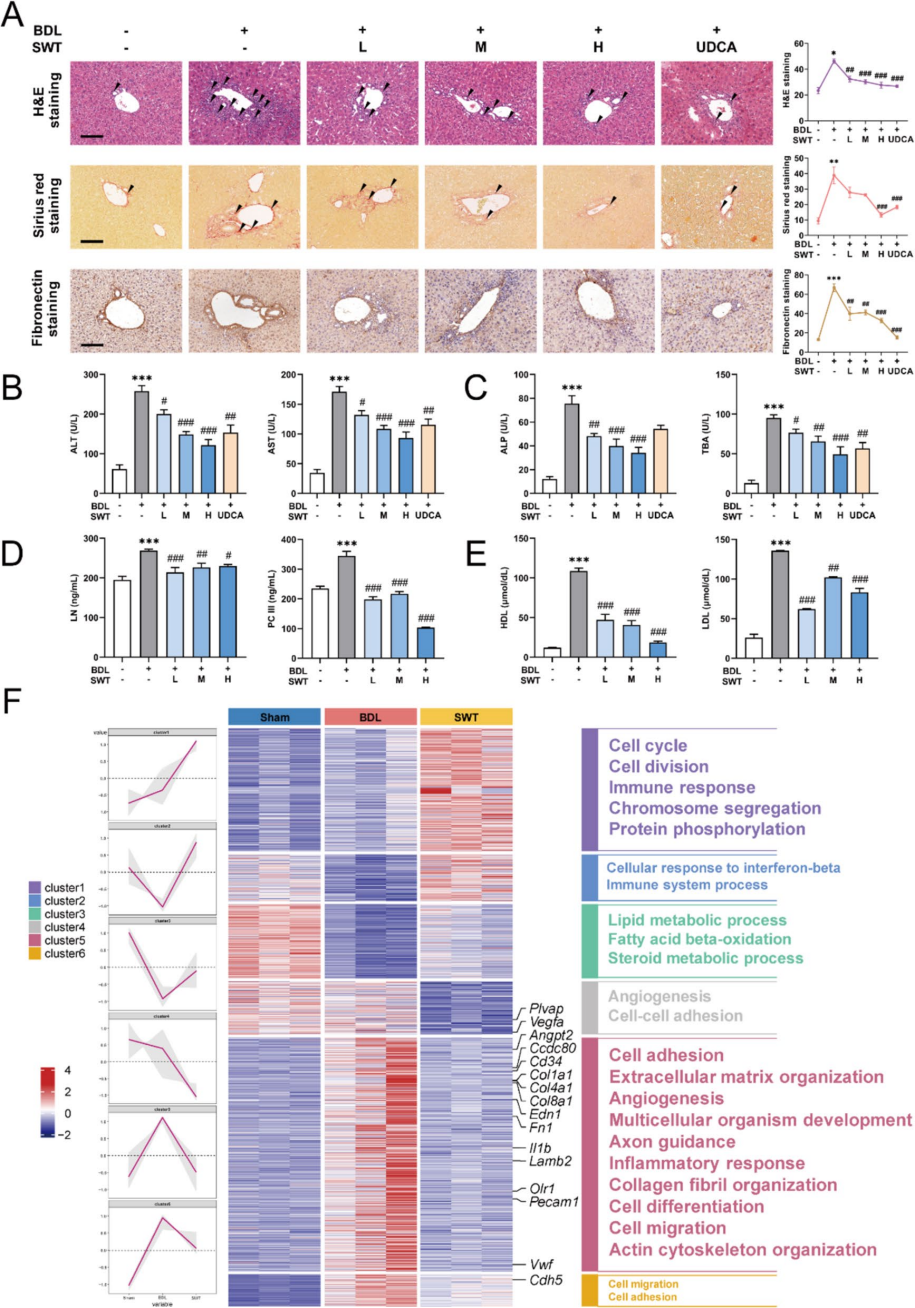
SWT significantly improves liver fibrosis induced by BDL in mice. **A** H&E, sirius red and fibronectin staining of the liver (scale bar = 100 μm). **B–E** Serum levels of ALT and AST (**B**), ALP and TBA (**C**), LN and PC III (**D**) as well as HDL and LDL (**E**). **F** Combination of trend graphs, heat maps and GO functional annotations based on gene sequencing data. Data are shown as mean ± SEM, n = 6 (One-way ANOVA with Tukey’s post-hoc tests). ***P < 0.001 as compared with the sham group; ^#^*P* < 0.05, ^##^*P* < 0.01, ^###^*P* < 0.001 as compared with the BDL group

To validate the therapeutic efficacy of SWT, H&E staining and sirius red staining were conducted on liver tissue samples to analyze their histopathological changes. As illustrated in Fig. 2A, the BDL group exhibited destruction of the hepatic portal area structure, increased collagen fiber deposition and significant inflammatory cell infiltration (black arrow), leading to a notable increase in liver fibrosis area, whereas SWT demonstrated a dose-dependent reversal of this process. Furthermore, the immunohistochemical staining results revealed that the distribution of the key marker of liver fibrosis, FN1 was significantly increased around hepatic portal areas in the BDL group, which was markedly reduced with SWT treatment. High dose of SWT showed improvements in liver fibrosis similar with UDCA. Further, we measured the expression of several hepatic injury markers in mouse serum. The results revealed significantly elevated expression of alanine aminotransferase (ALT), aspartate aminotransferase (AST), alkaline phosphatase (ALP) and total bile acid (TBA) in the serum of BDL group mice compared to the sham group, which were markedly reduced in a dose-dependent manner by SWT (Fig. 2B and C). SWT demonstrated liver damage repair effects akin to UDCA, with noteworthy superiority in ALP improvement over UDCA. At the same time, SWT markedly reduced the elevated expression of laminin (LN) and procollagen III (PC III) induced by BDL (Fig. 2D). We also assessed the levels of blood lipid-related markers high-density lipoprotein (HDL) and low-density lipoprotein (LDL) [32], and found that SWT significantly reduced the elevated blood lipids induced by BDL with a dose-dependent manner, indicating hepatic sinusoidal permeability was improved (Fig. 2E). To delve deeper into understanding the molecular mechanisms through which SWT ameliorates liver fibrosis, we subjected mouse liver samples to RNA-seq analysis. The ClusterGVis package was applied to analyze RNA-seq data, through which, we created a combined visualization of trend plots, heatmaps, and GO functional annotation, providing a comprehensive visual representation of the analysis results. As depicted in Fig. 2F, numerous genes are clustered around biological processes such as angiogenesis and cell adhesion. These genes significantly upregulated in the BDL group and markedly downregulated in the SWT group. Thus, we suggested that the amelioration of BDL-induced liver fibrosis by SWT might be attributed to the modulation of these pathological processes.

### SWT significantly inhibits LSEC angiogenesis in vivo and in vitro

**Fig. 3:**
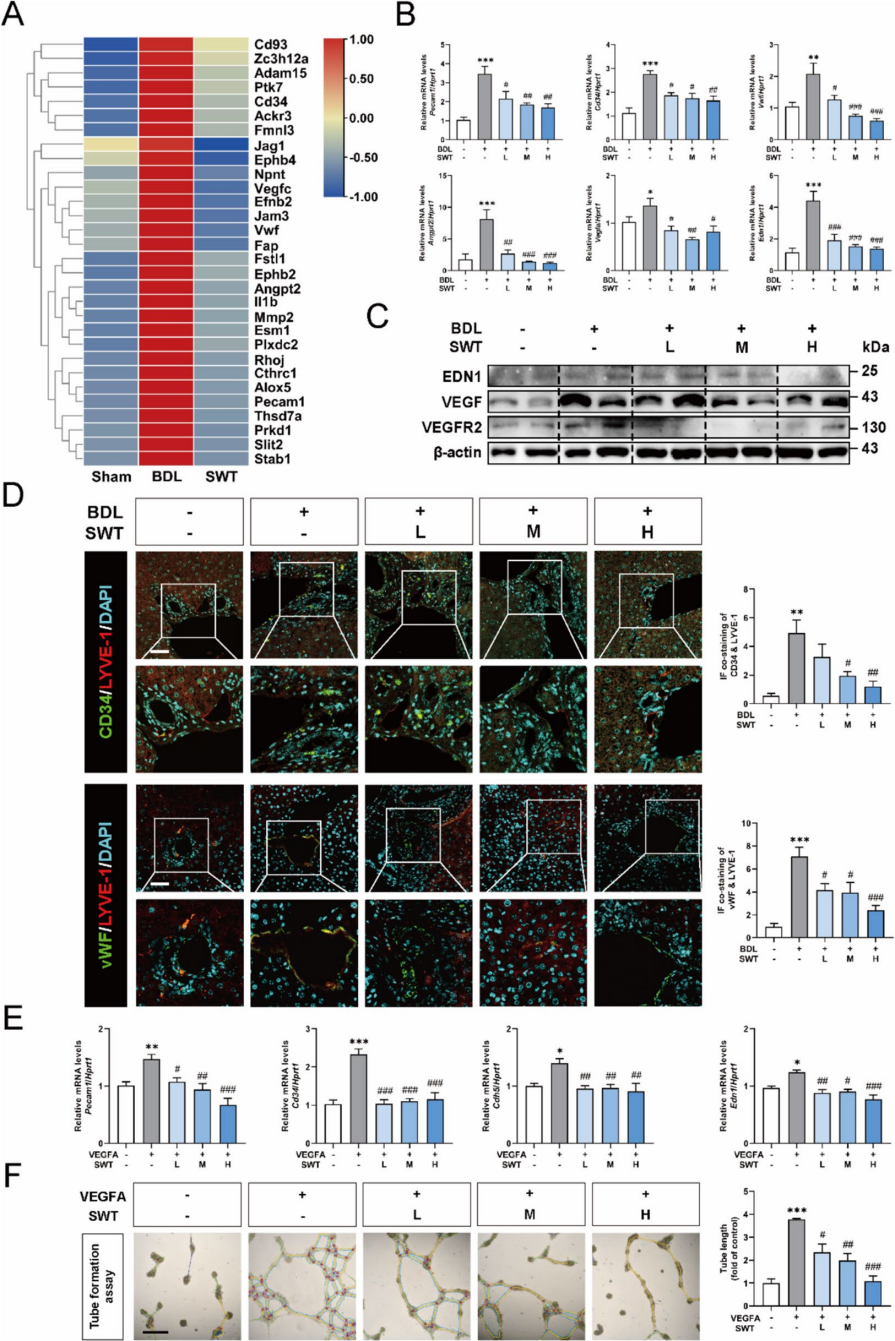
SWT inhibits LSEC angiogenesisin vivoandin vitro. **A** Heat map of genes related to angiogenesis. **B** The qPCR results of relative mRNA levels of *Pecam1*, *Cd34*, *Vwf*, *Angpt2*, *Vegfa*, and *End1* in mouse liver compared to *Hprt1*. **C** Western blot of relative protein expression levels of END1, VEGF, VEGFR2 in mouse liver compared to β-actin. **D** Immunofluorescence co-staining of CD34 or vWF with LYVE-1 in mouse liver (scale bar = 50 μm). **E** The qPCR results of relative mRNA levels of *Pecam1*, *Cd34*, *Cdh5* and *End1* in rat LSECs compared to *Hprt1*. **F** Tube formation experiment of LSECs (scale bar = 100 μm). Data are shown as mean ± SEM, n = 6 (One-way ANOVA with Tukey’s post-hoc tests). *P < 0.05, **P < 0.01, ***P < 0.001 as compared with the first group; ^#^*P* < 0.05, ^##^*P* < 0.01, ^###^*P* < 0.001 as compared with the second group

To delve deeper into the impact of SWT on angiogenesis, we crafted a heatmap depicting genes associated with vascular formation from the results of RNA-seq. As illustrated in Fig. 3A, compared to the sham group, the expression of angiogenesis-related genes was significantly upregulated in the BDL group, which was attenuated with SWT treatment. We further detected the expression of angiogenesis-related markers including platelet endothelial cell adhesion molecule (PECAM1) that also called CD31, CD34, vWF [34], pro-angiogenic cytokines angiopoietin-2 (ANGPT2), vascular endothelial growth factor A (VEGFA) [29], and endothelin-1 (END1) [39] in the mouse liver. The qPCR results demonstrated that SWT significantly reduces the overexpression of the aforementioned genes induced by BDL (Fig. 3B). Also, the effects of SWT on the level of most angiogenesis-related genes were dose-dependent. Simultaneously, western blot results demonstrated that SWT could attenuate the overexpression of pro-angiogenesis proteins such as END1, VEGFA and vascular endothelial growth factor receptor 2 (VEGFR2) in the mouse liver (Fig. 3C). Immunofluorescence co-staining of LYVE-1, the LSEC marker, along with angiogenesis markers CD34 or vWF demonstrated that BDL significantly increased angiogenesis in mouse LSEC and adjacent liver tissue, while SWT significantly reversed this process (Fig. 3D and Fig. S1A, B). Through bioinformatics analysis of the differentially expressed genes from the RNA-seq results, we discovered DEGs including the represented markers of undefined endothelial cell marker genes, indicating SWT might regulates LSECs, the liver specific endothelial cell (Fig. S2A). Moreover, the predicted data from The Human Protein Atlas showed that the angiogenesis-related gene were closely corrected with LSECs (Fig. S2B). To apply SWT in in vitro experiences, we first detected the impact of SWT on the cell viability of LSECs and found that 200 µg/ml SWT showed slightly inhibitory effect on rat LSECs (Fig. S2C). We selected the concentrations of 25 µg/ml, 50 µg/ml and 75 µg/ml for the in vitro studies. Subsequently, we discovered through colony formation assay that SWT exerted certain inhibitory effect on the proliferation of LSECs in vitro (Fig. S2D). The expression of pro-angiogenic genes such as *Pecam1*,* Cd34*,* Cdh5* [9] and *Edn1* in rat LSECs was assessed through qPCR. The results revealed that VEGFA significantly upregulated the expression of those genes, which exhibited noticeable reductions following treatment with different doses of SWT (Fig. 3E). Furthermore, the capability of SWT to inhibit angiogenesis was analyzed by in vitro tube formation assay. The results indicated that VEGFA could increase the tube-forming ability of LSECs, which was attenuated by SWT treatment with a dose-dependent manner (Fig. 3F). In summary, SWT effectively inhibited LSEC angiogenesis both in vivo and in vitro.

### SWT ameliorates LSEC adhesion by inhibiting basement membrane formation

**Fig. 4:**
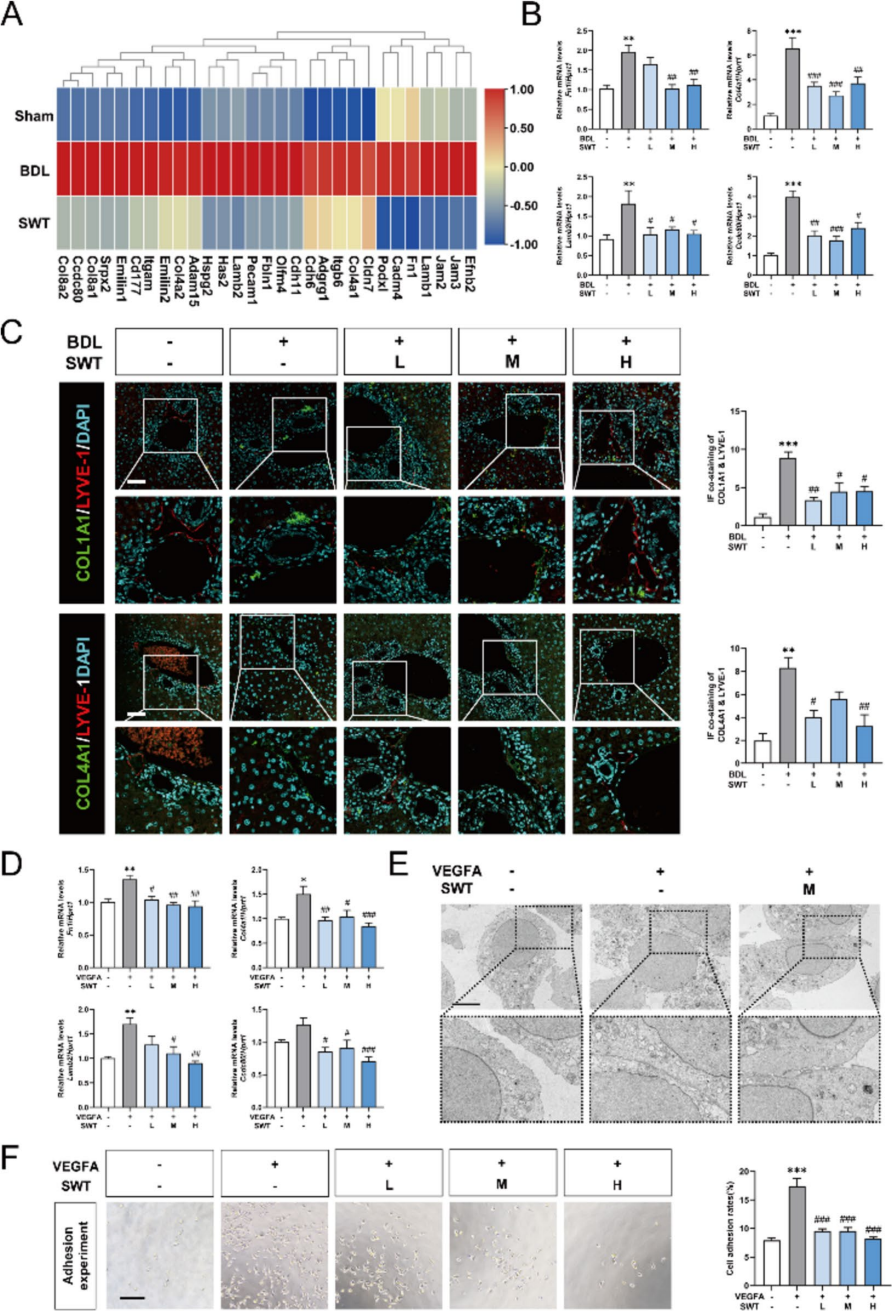
LSEC adhesion process is suppressed by SWTin vivoandin vitro. **A** Heat map of genes related to adhesion. **B** The qPCR results of relative mRNA levels of *Fn1*,* Col4a1*,* Lamb2* and *Ccdc80* in mouse liver compared to *Hprt1*. **C** Immunofluorescence co-staining of COL1A1 or COL4A1 with LYVE-1 in mouse liver (scale bar = 50 μm). **D** The qPCR results of relative mRNA levels of *Fn1*,* Col4a1*,* Lamb2* and *Ccdc80* in rat LSECs compared to *Hprt1*. **E** Transmission electron microscopy results of LSECs (scale bar = 5 μm). **F** Adhesion experiment of LSECs (scale bar = 100 μm). Data are shown as mean ± SEM, *n* = 6 (One-way ANOVA with Tukey’s post-hoc tests). *P < 0.05, **P < 0.01, ***P < 0.001 as compared with the first group; ^#^*P* < 0.05, ^##^*P* < 0.01, ^###^*P* < 0.001 as compared with the second group

Notably, in Fig. 2, we also mentioned that differentially expressed genes in RNA-seq of transcriptome were significantly enriched in the cell adhesion process. To further explore the impact of SWT on the generation of LSEC basement membrane and cell adhesion, firstly, we conducted heatmap to visualize adhesion and basement membrane-related genes that were up-regulated in BDL group and suppressed by SWT treatment (Fig. 4A). Then we employed qPCR and immunofluorescence staining to validate the expression of cell adhesion-promoting proteins FN1 [10] and CCDC80 [25], and basement membrane factors collagen type IV alpha 1 (COL4A1), COL1A1 and LAMB2 [4] in the mouse liver. Subsequent qPCR analysis validated those genes such as *Fn1*,* Col4a1*,* Lamb2* and *Ccdc80* were up-regulated in the BDL group, while SWT treatment significantly inhibited these up-regulation genes (Fig. 4B). Immunofluorescence staining indicated that SWT significantly reversed the basement membrane formation induced by BDL in the LSECs and hepatic sinusoidal regions (Fig. 4C and Fig. S3A-C). The qPCR results of the in vitro experiments also confirmed the results of the in vivo experiments (Fig. 4D). Furthermore, we conducted transmission electron microscopy scanning on cultured LSECs and found that, compared to the control group, cell-cell connections were increased in a VEGFA-simulated liver fibrotic environment, which was partially alleviated by SWT (medium dosage) treatment (Fig. 4E). Further cell adhesion experiments confirmed the inhibitory effect of SWT on VEGFA-induced adhesion process (Fig. 4F). These results suggested that SWT could improve cell adhesion by inhibiting the formation of LSEC basement membrane and tight junctions between cells.

### LSEC fenestration is restored by SWT through cellular cytoskeleton remodeling

**Fig. 5:**
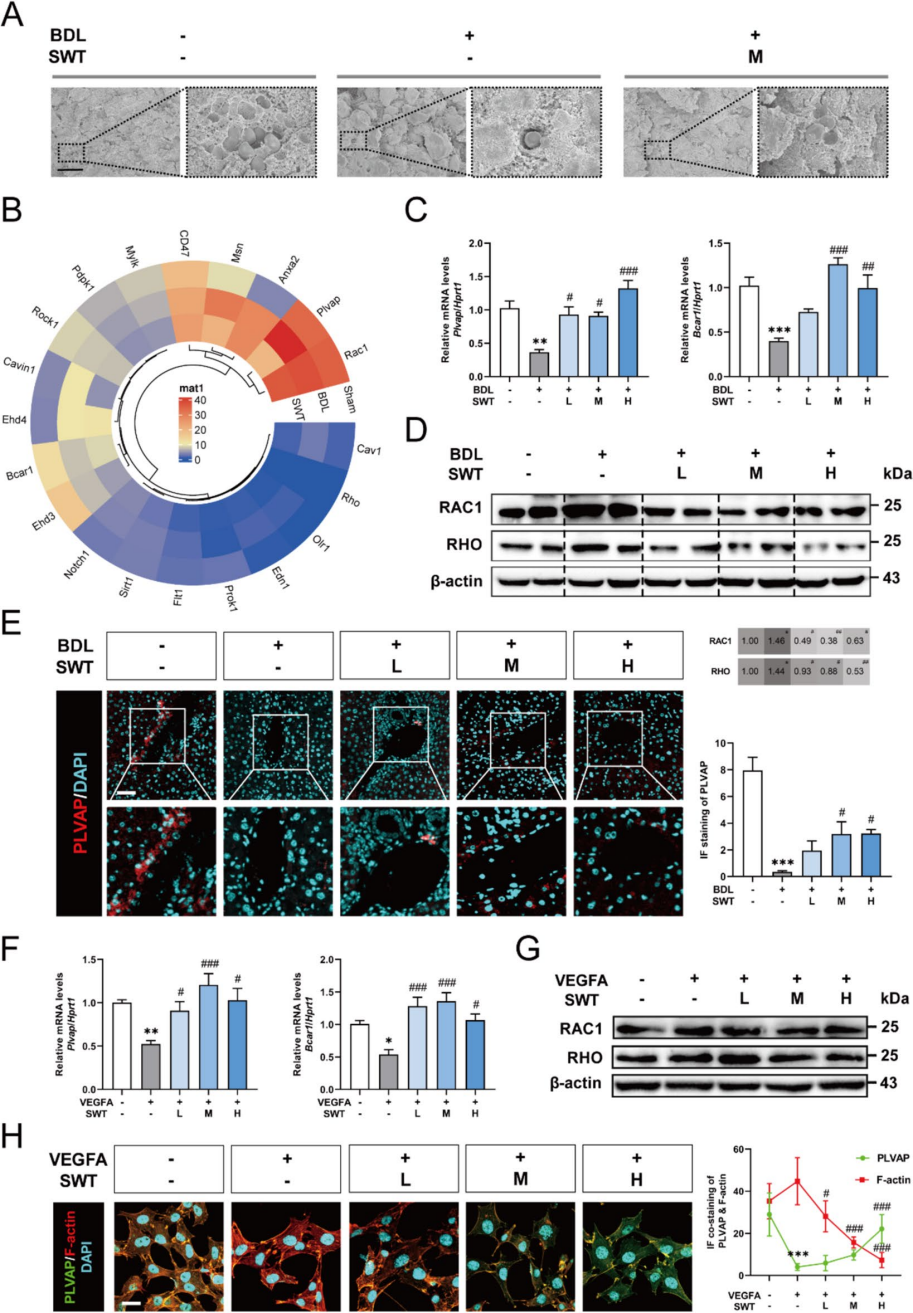
SWT restores LSEC fenestration by regulating cytoskeletal remodeling. **A** Scanning electron microscopy results of mouse liver (scale bar = 25 μm). **B** Heat map of genes related to fenestrae. **C** The qPCR results of relative mRNA levels of *Plvap* and *Bcar1* in mouse liver compared to *Hprt1*. **D** Western blot of relative protein expression levels of RAC1 and RHO in mouse liver compared to β-actin. **E** Immunofluorescence of PLVAP in mouse liver (scale bar = 50 μm). **F** The qPCR results of relative mRNA levels of *Plvap* and *Bcar1* in rat LSECs compared to *Hprt1*. **G** Western blot of relative protein expression levels of RAC1 and RHO in rat LSECs compared to β-actin. **H** Immunofluorescence co-staining of PLVAP & F-actin in rat LSECs (scale bar = 20 μm). Data are shown as mean ± SEM, n = 6 (One-way ANOVA with Tukey’s post-hoc tests). *P < 0.05, **P < 0.01, ***P < 0.001 as compared with the first group; ^#^*P* < 0.05, ^##^*P* < 0.01, ^###^*P* < 0.001 as compared with the second group

As a hallmark phenotypic feature of LSEC dedifferentiation, there is a significant reduction in LSEC fenestration during liver fibrosis [11]. The results obtained from scanning electron microscopy revealed that the sham group exhibited a distinct patchy distribution of fenestrae in the hepatic sinusoid areas. In contrast to the sham group, there was a significant reduction in the number of fenestrae within the hepatic sinusoids of the BDL group. However, following SWT treatment, a partial restoration of fenestrae in the hepatic sinusoid was observed (Fig. 5A). A heatmap was generated to demonstrate the effects of SWT on the fenestrae-regulating genes identified in the RNA-seq results when compared with BDL group (Fig. 5B). Previous studies have reported the crucial role of PLVAP in the formation of LSEC fenestrae and its modulation by the actin cytoskeleton [14]. Meanwhile, another research has highlighted the positive regulatory role of breast cancer anti-estrogen resistance protein 1 (BCAR1) in LSEC fenestrae [31]. Through qPCR, we discovered that genes associated with fenestration, *Plvap* and *Bcar1*, were downregulated after BDL but restored to an upregulated state following SWT treatment (Fig. 5C). As reported, Rac1 and Rho, the pivotal upstream factors regulating the actin cytoskeleton could induce the constriction and the defenestration of LSEC fenestrae [37]. Therefore, we further detected the protein expression of Rac1 and Rho and found the inhibitory effects of SWT on the BDL-induced overexpression of these proteins (Fig. 5D). The immunofluorescence staining results also unveiled the role of PLVAP and BCAR1 in LSEC fenestration and the restoring effects of SWT (Fig. 5E and Fig. S4A, B). Moreover, we conducted in vitro studies and found that the qPCR results, as shown in Fig. 5F, exhibited similar outcomes to those observed in vivo. The results of cellular western blot analysis were consistent with those observed in mouse liver tissues (Fig. 5G). As the function of cytoskeletal protein F-actin in LSEC fenestrae, we conducted immunofluorescence co-staining of F-actin and PLVAP. In Fig. 5H and Fig. S4C, VEGFA induced a reduction in LSEC fenestrae, while SWT promoted the restoration of LSEC fenestrae. At the same time, SWT promoted the depolymerization of the F-actin. Consider that, we suspended that SWT modulated cytoskeleton remolding was positively contributed to the reformation of LSEC fenestrae.

### SWT improves liver fibrosis by modulating angiogenesis, cell adhesion and fenestration through the COL8A1/IL-1β/OLR1 pathway

**Fig. 6:**
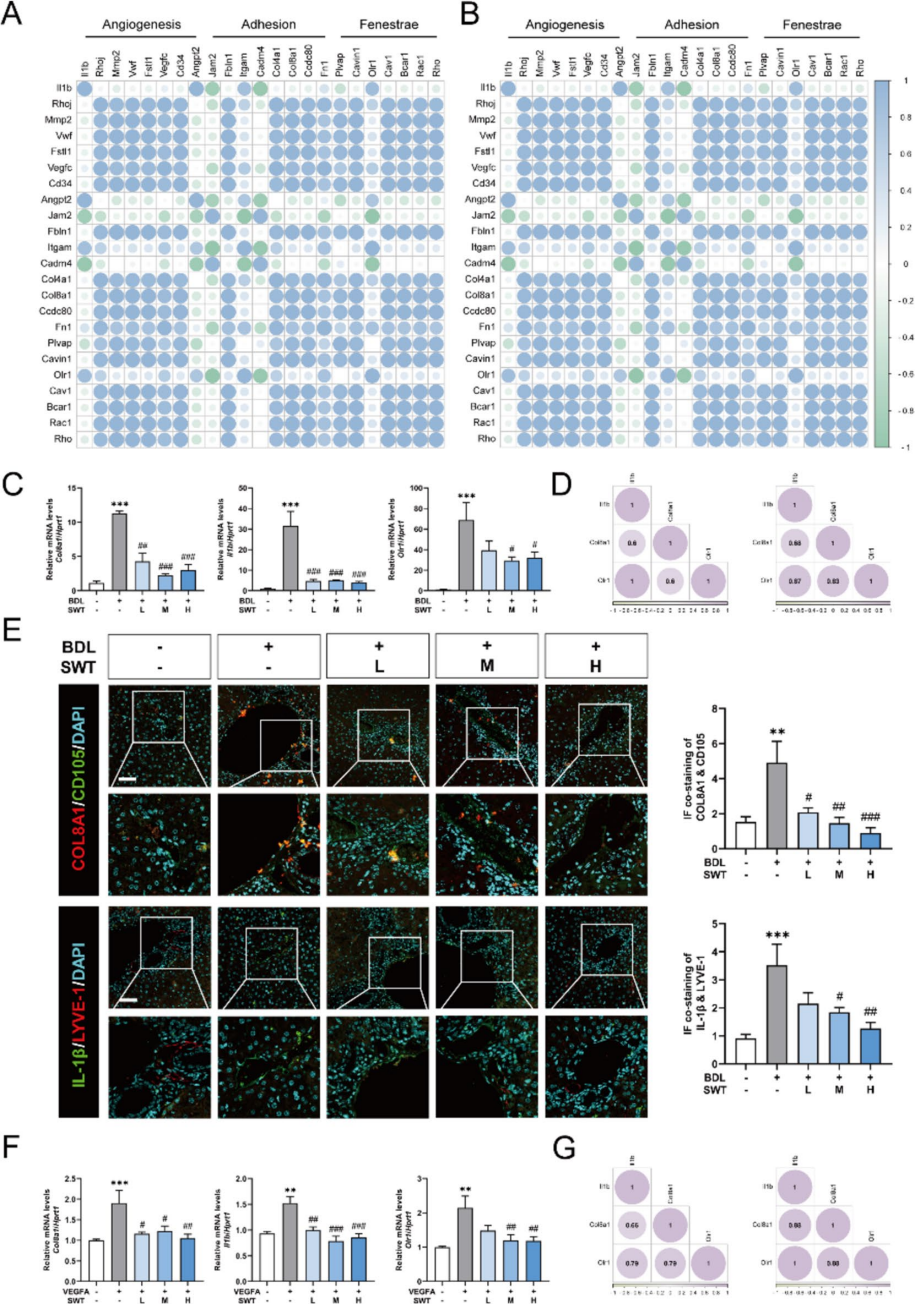
Regulation of COL8A1, IL-1β, and OLR1 by SWT and correlation analysis among them. **A**,** B** Correlation analysis of *Col8a1*, *Il1b* and *Olr1* in mice of the BDL group (**A**) and the BDL + SWT group (**B**). **C** The qPCR results of relative mRNA levels of *Col8a1*,* Il1b* and *Olr1* in mouse liver compared to *Hprt1*. **D** Correlation validation of in vivo experiments. **E** Immunofluorescence co-staining of COL8A1 & CD105 and IL-1β & LYVE-1 in mouse liver (scale bar = 50 μm). **F** The qPCR results of relative mRNA levels of *Col8a1*,* Il1b* and *Olr1* in rat LSECs compared to *Hprt1*. **G** Correlation validation of in vitro experiments. Data are shown as mean ± SEM, n = 6 (One-way ANOVA with Tukey’s post-hoc tests). *P < 0.05, **P < 0.01, ***P < 0.001 as compared with the first group; ^#^*P* < 0.05, ^##^*P* < 0.01, ^###^*P* < 0.001 as compared with the second group

In order to delve deeper into the mechanisms by which SWT regulated the angiogenesis, adhesion and fenestration of LSECs during hepatic fibrosis, we employed spearman algorithms to conduct correlation coefficient analysis on genes associated with angiogenesis, adhesion and fenestration based on the RNA-seq results. Notably, the results revealed a significant positive correlation between *Il1b* and oxidized low-density lipoprotein receptor 1 (*Olr1*) in the BDL group (Fig. 6A). Besides, in Fig. 6B, we demonstrated a conspicuous positive correlation among *Col8a1*, *Il1b* and *Olr1* in the BDL + SWT group, indicating theses gene were highly related especially with SWT treatment. We further confirmed the impact of SWT on the mRNA and protein levels of these genes through qPCR and immunofluorescence. As depicted in Fig. 6C, in the mouse BDL model, the qPCR results showed that SWT markedly attenuated the mRNA expression of *Col8a1*, *Il1b*, and *Olr1* induced by BDL. Additionally, in both the BDL and BDL + SWT groups, the mRNA expression of *Col8a1*, *Il1b*, and *Olr1* demonstrated a robust correlation (Fig. 6D). Immunofluorescence staining of mouse liver tissue revealed enhanced expression of COL8A1 and IL-1β in LSECs and adjacent areas in the BDL group, gradually downregulated upon SWT treatment (Fig. 6E and Fig. S5A, B). Similarly, we obtained comparable qPCR and correlation coefficient validation results in the VEGFA-induced cell model (Fig. 6F, G).

**Fig. 7:**
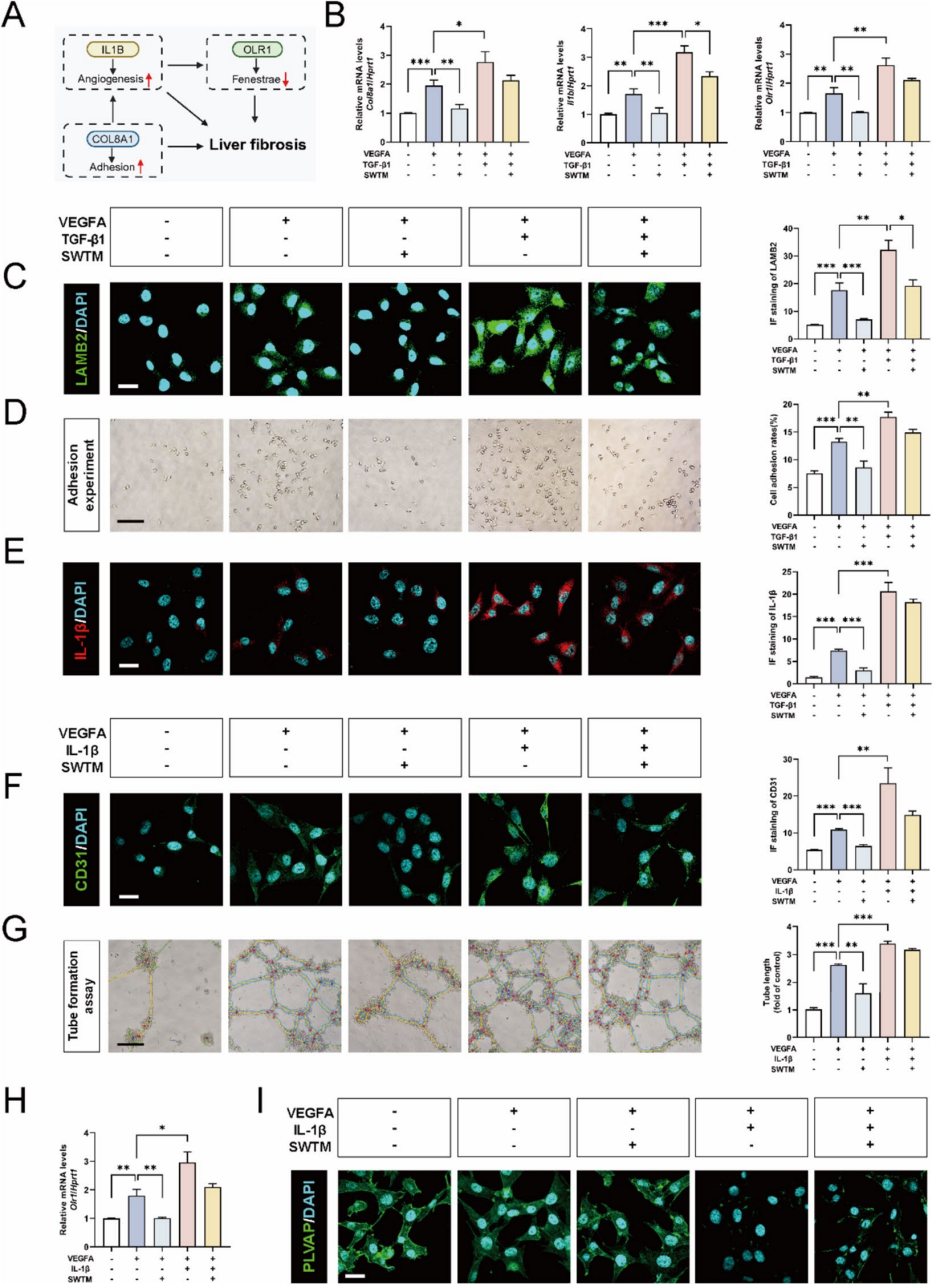
SWT inhibits the COL8A1/IL-1β/OLR1 pathway in LSECs. **A** The signaling diagram including COL8A1, IL-1β and OLR1. **B** The qPCR results of relative mRNA levels of *Col8a1*,* Il1b* and *Olr1* in rat LSECs compared to *Hprt1*. **C** Immunofluorescence staining of LAMB2 in rat LSECs (scale bar = 20 μm). **D** LSEC adhesion detection (scale bar = 100 μm). **E**,** F** Immunofluorescence staining of IL-1β (**E**) and CD31 (**F**) in rat LSECs (scale bar = 20 μm). **G** Tube formation experiment of LSECs (scale bar = 100 μm). **H** The qPCR results of relative mRNA levels of *Olr1* in rat LSECs compared to *Hprt1*. (**I**) Immunofluorescence staining of PLVAP in rat LSECs (scale bar = 20 μm). Data are shown as mean ± SEM, n = 6 (One-way ANOVA with Tukey’s post-hoc tests). *P < 0.05, **P < 0.01, ***P < 0.001 as compared with another group

Prior studies have reported that COL8A1 could induce the expression of IL-1β [28], which further promoted the expression of OLR1 [18]. Meanwhile, it was reported that COL8A1 could promote cellular adhesion process [1], while IL-1β could induce pathological angiogenesis [24]. Besides, fenestration formation was inhibited by OLR1 [38]. Therefore, we hypothesized that the network illustrated in Fig. 7A might serve a critical function in LSEC dedifferentiation. To further validate this hypothesis, we activated COL8A1 in LSECs in vitro by treating with recombinant rat TGF-β1 (10 ng/ml) [20]. The results revealed a further upregulation of *Col8a1* in the VEGFA-induced cell model, leading to subsequent upregulation of downstream *Il1b* and *Olr1*. It’s noteworthy that the upregulation of *Col8a1* partially reversed the efficacy of SWT (Fig. 7B). Cellular immunofluorescence staining demonstrated that TGF-β1 additionally boosted the expression of adhesive marker LAMB2 following VEGFA stimulation, while partially counteracting the inhibitory impact of SWT on the adhesion process (Fig. 7C and Fig. S6A). Cell adhesion experiments also illustrated the bidirectional regulatory effects of TGF-β1 and SWT on the adhesion process (Fig. 7D). Besides, immunofluorescence staining also revealed that COL8A1 was the upstream signal of IL-1β (Fig. 7E and Fig. S6B). Then, we employed recombinant rat IL-1β to reverse the downregulation of IL-1β induced by SWT and found that SWT inhibited angiogenesis process that characterized by CD31 expression (Fig. 7F and Fig. S6C). Additional angiogenesis assays also unveiled that IL-1β not only promoted LSEC angiogenesis, but also reduced the inhibition of SWT on angiogenesis (Fig. 7G). Furthermore, the qPCR results revealed the ability of IL-1β to stimulate the expression of *Il1b* and downstream *Olr1* (Fig. 7H and Fig. S6D). Subsequent immunofluorescence staining revealed that IL-1β reversed the restorative effect of SWT on the LSEC fenestration marker PLVAP, indirectly affirming OLR1 as a downstream signal of IL-1β. (Fig. 7I and Fig. S6E). Taken together, these findings suggested that SWT might ameliorate hepatic fibrosis by potentially inhibiting the adhesive process mediated by COL8A1, the angiogenesis process mediated by IL-1β and the LCEC defenestration mediated by OLR1. Also, the upstream and downstream relationships of these three key genes were validate.

### SWT reverses HSC activation by inhibiting IL-1β secretion from LSECs

**Fig. 8:**
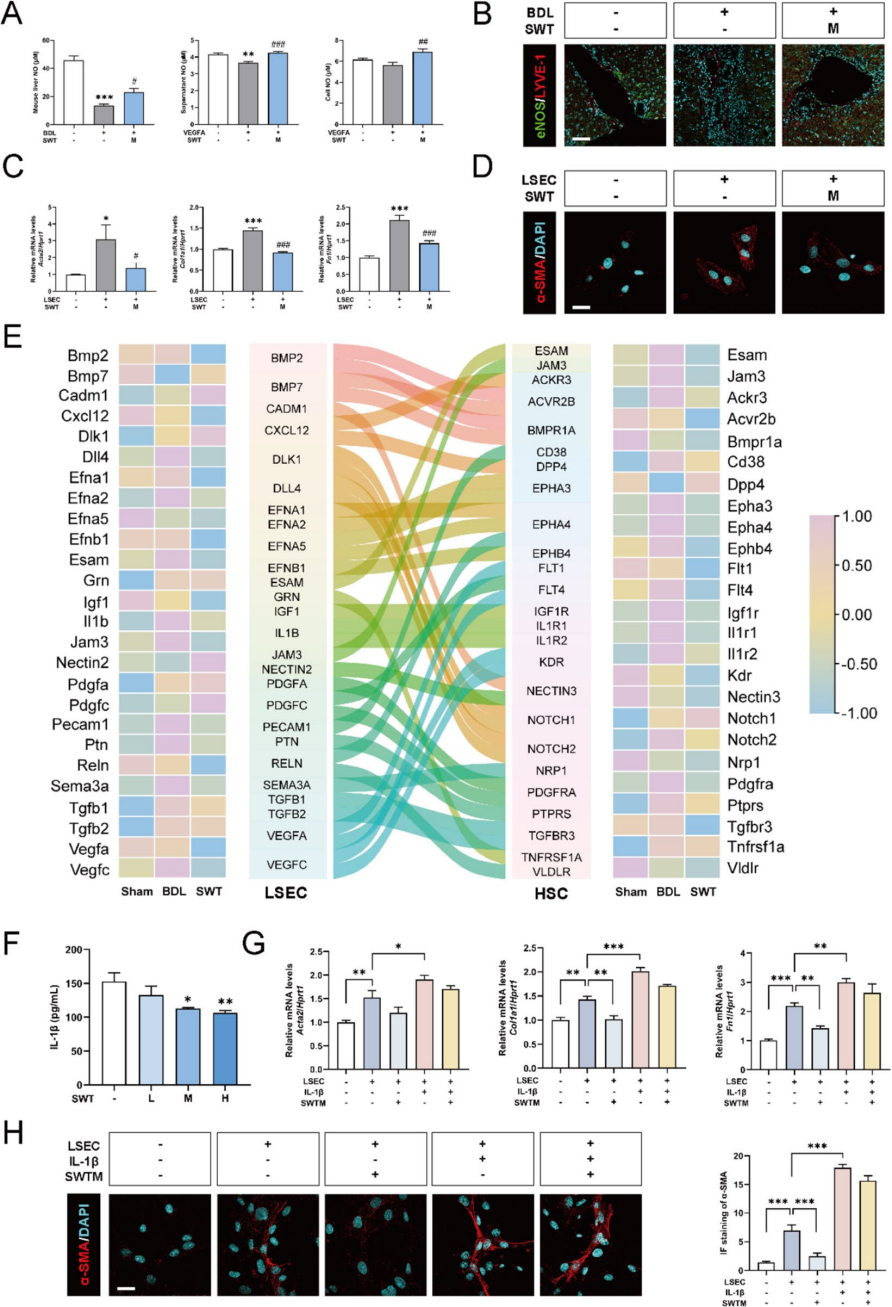
Improvement of liver fibrosis by regulating the communication between LSECs and HSCs***via***SWT. **A** Detection of NO content. **B** Immunofluorescence co-staining of eNOS & LYVE-1 in mouse liver (scale bar = 50 μm). **C** The qPCR results of relative mRNA levels of *Acta2*,* Col1a1* and *Fn1* in rat HSC-T6 compared to *Hprt1*. **D** Immunofluorescence staining of α-SMA in rat HSC-T6 (scale bar = 20 μm). **E** Network diagram of ligand-receptor interaction between LSECs and HSCs along with the heat map of gene expression. **F** Detection of IL-1β content in the supernatant of LSEC culture. **G** The qPCR results of relative mRNA levels of *Acta2*,* Col1a1* and *Fn1* in rat HSC-T6s compared to *Hprt1*. **H** Immunofluorescence staining of α-SMA in rat HSC-T6 (scale bar = 20 μm). *P < 0.05, **P < 0.01, ***P < 0.001 as compared with the first group; ^#^*P* < 0.05, ^##^*P* < 0.01, ^###^*P* < 0.001 as compared with the second group

It was well known that endothelial nitric oxide synthase (eNOS) regulated the secretion of NO in LSECs, which played a crucial role in maintaining the quiescence of HSCs. Therefore, we measured NO levels in mouse liver, rat LSECs, and LSEC supernatants separately and found that NO level was decreased in both the in vivo BDL model and in vitro VEGFA model, which was increased with SWT treatment. Although the decrease in NO content in the cell supernatant did not reach statistical significance, the trend of NO change was still consistent with other results (Fig. 8A). Animal fluorescence imaging further demonstrated the downregulation of eNOS in LSECs and adjacent hepatic sinusoidal regions induced by BDL, which was partially reversed by SWT (Fig. 8B and Fig. S7A). To further explore the role of LSECs in HSCs, we applied the conditioned media from VEGFA-treated rat LSECs to rat HSC-T6 cells. We found that compared to the control group, LSEC supernatant treatment directly upregulated the expression of activation-related genes in HSCs, such as *Acta2*, *Col1a1*, and *Fn1*, while the supernatant of LSECs with SWT administration partially reversed HSC activation (Fig. 8C). Immunofluorescence staining for α-SMA further confirmed these results (Fig. 8D and Fig. S7B). As shown in Fig. 8E, previous studies have reported the interplay between LSECs and HSCs through interactions mediated by different ligand receptors [21]. Among these factors, several researches demonstrated that IL-1β played a crucial role in the communication among various hepatic cells [2]. We have found that SWT could significantly inhibit the release of IL-1β from LSECs. Despite it was widely known that IL-1β could activate HSCs, it was still unknown whether IL-1β released from LSECs could contribute to promoting HSC activation through the paracrine pathway. Hence, we further examined the IL-1β content in LSEC supernatant and found that SWT dose-dependently inhibited IL-1β expression in LSECs (Fig. 8F). Further, we added recombinant rat IL-1β to the culture media of LSEC with the administration of SWT. After culturing HSCs with the conditioned medium of LSECs, both qPCR and immunofluorescence results of HSCs indicated that IL-1β could further activate HSCs in vitro, and partially reverse the inhibitory effects of SWT on HSC activation (Fig. 8G, H and Fig. S7C). In summary, IL-1β of LSECs could activate HSCs through a paracrine pathway to promote hepatic fibrosis, while SWT significantly reversing this process.

## Discussion

SWT, as a classic formula, was originally not used in equal proportions. Instead, it featured Shudihuang, Danggui, Baishao and Chuanxiong as the monarch, minister, assistant and envoy herbs, respectively, which was widely applied in the clinical treatment of various gynecological conditions. In recent years, there has been growing interests to employ SWT in equal proportions for the treatment of hepatic fibrosis and other chronic liver diseases, yielding promising results [23, 27, 35]). SWT achieved its remarkable anti-fibrotic effects through multiple targets and pathways, but the multiple mechanism underlying SWT remains to be fully elucidated. Hence, this study focused on LSECs, the predominant non-parenchymal cells within the liver, to investigate their pathological changes during liver fibrosis and their interactions with HSCs. Initially, SWT was prepared following standard procedures, and following mass spectrometry analysis was conducted (Fig. 1). Subsequently, we established BDL-induced mice model of liver fibrosis and applied RNA-seq combined with bioinformatic analysis to uncover the potential pathological processes modulated by SWT (Fig. 2). Specifically, SWT improved liver fibrosis by inhibiting LSEC angiogenesis, adhesion processes and defenestration (Figs. 3, 4 and 5). Furthermore, additional validations were performed on the adhesive promotion by COL8A1, angiogenic effects facilitated by IL-1β, OLR1-induced LSEC defenestration, as well as the regulatory impacts of SWT on the COL8A1/IL-1β/OLR1 pathway (Figs. 6 and 7). Of note, SWT could also reverse HSC activation through LSECs, thereby alleviating liver fibrosis (Fig. 8).

Due to the observed alterations in biological processes such as angiogenesis and cell adhesion in RNA-seq in the SWT group, we speculated that SWT might significantly modulate LSEC functions, thereby ameliorating liver fibrosis and initiating subsequent validation. Li et al. [17] reported a therapeutic strategy involving the restoration of LSECs by riociguat to alleviate liver fibrosis, indicating LSECs was a potential cell target. In this study, we have unveiled, for the first time, the regulatory effects of SWT on various dedifferentiation processes within LSECs, encompassing angiogenesis, basement membrane formation, and defenestration. During liver fibrosis, given that LSEC angiogenesis is a common observation, extensive research has documented the alleviation of liver fibrosis through pharmaceutical interventions targeting LSEC angiogenesis [36]. Similarly, this study robustly confirmed the inhibitory effects of SWT on LSEC angiogenesis, which was validated in vivo and in vitro (Fig. 3). It was reported that the downregulation of vascular cell adhesion molecule 1 expression associated with LSECs could inhibit the cell adhesion process, thereby alleviating liver fibrosis [3, 13]. Moreover, based on the significance of LSEC fenestration and the lack of research in this area, we further investigated the modulation of SWT on this process. Consistent with previous findings of reduced LSEC fenestration in liver fibrosis, our findings also indicated a significant reduction of LSEC fenestrae in liver fibrosis, while SWT aided in the restoration of fenestration. Moreover, Wei et al. have demonstrated that the depolymerization of F-actin promoted the restoration of LSEC fenestrations, elucidating the dynamic interplay between cellular cytoskeletal dynamics and fenestration formation [33]. In initial findings, we noted that SWT notably intervened in the expression of cytoskeletal-related genes within the liver during fibrosis. However, does it impact the cytoskeleton in LSECs? Did this process correlate with the formation of LSEC fenestrations? These questions were explored in our study. It is noteworthy that, during the process of LSEC fenestration regeneration, there was concurrent disassembly and reorganization of the cellular cytoskeleton (Fig. 5). After undergoing processes such as vascularization, increased adhesion, and decreased fenestration, LSECs significantly reduced liver sinusoidal permeability. Li et al. supported that restoring hepatic sinusoidal permeability during liver fibrosis not only helped the maintain of basic liver physiological functions but also enhanced drug absorption efficiency [17]. It is worth noting that BDL induced a significant increase in serum levels of HDL and LDL, while a process significantly reversed by SWT (Fig. 2D). The reduced hepatic sinusoidal permeability induced by BDL could lead to a decrease in the hepatic metabolism of HDL and LDL, consequently causing their accumulation in the circulatory system, which significantly ameliorated by SWT. With experimental validation, we suggested that the improvement in liver sinusoidal permeability was attributed to the modulation of SWT on multiple biological processes of LSECs.

These three main biological processes of LSECs we mentioned were potential connected. Several researches have reported that during the progression of liver fibrosis, cellular adhesion involved LSECs was closely connected with hepatic capillarization [15]. Yet, there is a paucity of research exploring the association among LSEC fenestration and these two processes. Hence, we conducted experiments that aimed to reveal the potential mechanisms by which SWT regulated the three pathological processes: angiogenesis, cell adhesion and defenestration (Fig. 6). Previous researches have suggested that COL8A1, IL-1β and OLR1 were involved in the process of liver fibrosis. However, the functions of COL8A1 and OLR1 in LSECs during liver fibrosis were not clear. In our study, based on the high connection of these three genes, we explored the regulation of SWT on the three genes and found a noteworthy inhibition of their expression. We systematically validated the adhesive role of COL8A1, the impact of IL-1β on angiogenesis and the negative regulation of fenestrae *via* OLR1 in LSECs. Also, we were the first to identify the upstream-downstream relationship of the three genes in LSECs, which was corroborated by the work of other researchers [18, 28]. For example, it has been reported that the overexpression of COL8A1 markedly enhanced IL-1β expression, while treatment with recombinant rat COL8A1 could reverse the downregulation of IL-1β expression caused by COL8A1 knockout ^16^. Additionally, IL-1β was reported to promote the expression of OLR1 mRNA and protein levels in a time- and dose-dependent manner ^17^. Furthermore, it is worth discussing that SWT, as a compound formulation, could function through multiple targets and pathways. Our previous studies have reported that SWT might improve liver fibrosis by regulating the expression of Myo1c, Syde1 and Rhoj in LSECs. This might explain why inducing COL8A1 overexpression or supplementing IL-1β protein could not completely reverse the therapeutic effects of SWT (Fig. 7).

In the Disse space, LSECs and HSCs are in close physical proximity, fostering intimate interactions. It was widely acknowledged that LSECs played a gatekeeper in maintaining HSC quiescence under physiological conditions. Nevertheless, once LSECs undergo dedifferentiation, they produced less NO, resulting in losing the inhibition on HSCs activation. In this study, we conducted intercellular communication experiments between rat LSECs and rat HSCs to ensure the homogeneity of these two types of cells. Also, SWT significantly inhibited the expression of IL-1β. Moreover, as a secretory protein, the content of IL-1β that secreted from LSECs in the extracellular space was significantly decreased concurrently (Fig. 8F). Importantly, it has been reported that IL-1β administration could stimulate the expression of ROS, α-SMA and collagen III proteins, as well as activate NF-κB in HSCs [5]. Based on previous research as well as our RNA-seq analysis and experimental results, we supported that LSECs might facilitate HSC activation through the paracrine releasing of IL-1β, whereas SWT restored HSC quiescence by inhibiting IL-1β secretion in LSECs. These findings not only complemented prior researches on the interaction of IL-1β among different liver cells but also unveiled the inhibitory role of SWT on the releasing of IL-1β from LSECs in a paracrine manner.

## Conclusion

Overall, our findings suggested that SWT restored liver sinusoidal permeability and alleviated the progression of liver fibrosis by inhibiting COL8A1/IL-1β/OLR1 conducted LSEC angiogenesis, adhesion and defenestration. Furthermore, LSEC-mediated activation of HSCs was suppressed by SWT *via* inhibiting the releasing of IL-1β from LSECs. These findings enhanced the understanding of the pharmacological mechanism by which SWT targeted LSEC cells to exert its anti-liver fibrosis effects.

## Supplementary Information

Below is the link to the electronic supplementary material.

Supplementary Material 1

## Data Availability

All data included in this article are available from the corresponding author.

## Abbreviations

ALP: Alkaline phosphatase
ALT: Alanine aminotransferase
*ANGPT2*: Angiopoietin-2
AST: Aspartate aminotransferase
BCAR1: Breast cancer anti-estrogen resistance protein 1
BDL: Bile duct ligation
CCDC80: Coiled-coil domain-containing protein 80
COL4A1: Collagen type IV alpha 1
COL1A1: Collagen type I alpha 1
COL8A1: Collagen type VIII alpha 1
ECM: Extracellular matrix
END1: Endothelin-1
eNOS: Endothelial nitric oxide synthase
FN1: Fibronectin
GO: Gene Ontology
HSC: Hepatic stellate cell
HDL: High-density lipoprotein
LAMB2: Laminin beta-2
LDL: Low-density lipoprotein
IL-1β: Interleukin-1 beta
LN: Laminin
LSEC: Liver sinusoidal endothelial cell
NO: Nitric oxide
OLR1: Oxidized low-density lipoprotein receptor 1
PCIII: Procollagen III
PLVAP: Plasmalemma vesicle-associated protein
SWT: Si-Wu-Tang
TBA: Total bile acid
UHPLC: Ultra-high-performance liquid chromatography
VEGF: Vascular endothelial growth factor
VEGFR2: Vascular endothelial growth factor receptor 2
vWF: von Willebrand factor
