# Supplementary material for: Si-Wu-Tang improves liver fibrosis by restoring liver sinusoidal endothelial cell functionality and reducing communication with hepatic stellate cells
Source: Chin Med. 2024 Dec 31;19:179. doi: 10.1186/s13020-024-01038-1 (PMC11686876; doi:10.1186/s13020-024-01038-1)
Supplement: Supplementary file 1 — Supplementary Material 1 [file 13020_2024_1038_MOESM1_ESM.docx]

**Supplementary files**

**1. Supplementary methods**

**1.1 Serum biochemistry**

After placed for 1 h, the serum was extracted from blood. Refer to the instructions provided with the kits, the levels of alanine aminotransferase (ALT), aspartate aminotransferase (AST), alkaline phosphatase (ALP), total bile acid (TBA), laminin (LN), procollagen III (PC III), high-density lipoprotein (HDL) and low-density lipoprotein (LDL) in the serum were measured. ALT (C009-2-1), AST (C010-2-1), ALP (A059-2-2) and TBA (E003-2-1) kit were obtained from Nanjing Jiancheng Bioengineering Institute Co., Ltd. China. Mouse LN ELISA kit (H148) and mouse PC III ELISA kit (H212) were obtained from Nanjing Jiancheng Bioengineering Institute Co., Ltd. China. HDL kit (BC5325) and LDL kit (BC5335) were obtained from Beijing Solarbio Science & Technology Co., Ltd. China.

**1.2 Liver histopathology**

As for liver histopathological staining, partial liver tissue was fixed in formalin solution, embedded in paraffin, sectioned continuously, stained with hematoxylin-eosin (H & E), and sirius red, then observed for pathological changes under the A009 super-resolution microscopy tissue imaging system (Leica Aperio Versa).

**1.3 Quantitative RT-PCR**

Total RNA was extracted from mouse liver using trizol reagent, and cDNA was synthesized for qRT-PCR analysis. Hprt1 was selected as an internal reference gene, and the expression of the target gene was calculated based on the ratio of the target gene to the Hprt1. For further information on the specific primer sequences, please reach out to the corresponding author.

**1.4 Western blot analysis**

We dissolved mouse liver tissue or rat LSECs in RIPA lysis buffer containing phosphatase and protease inhibitors and determined the protein concentration through the BCA protein assay kit (Biorigin, Beijing, China). The expression level of the target protein was assessed based on the intensity of the bands, adjusting the sample load until the internal reference protein β-actin showed identical expression levels. Finally, the expression levels of the target protein were compared among different groups.

**2. Supplementary figures**


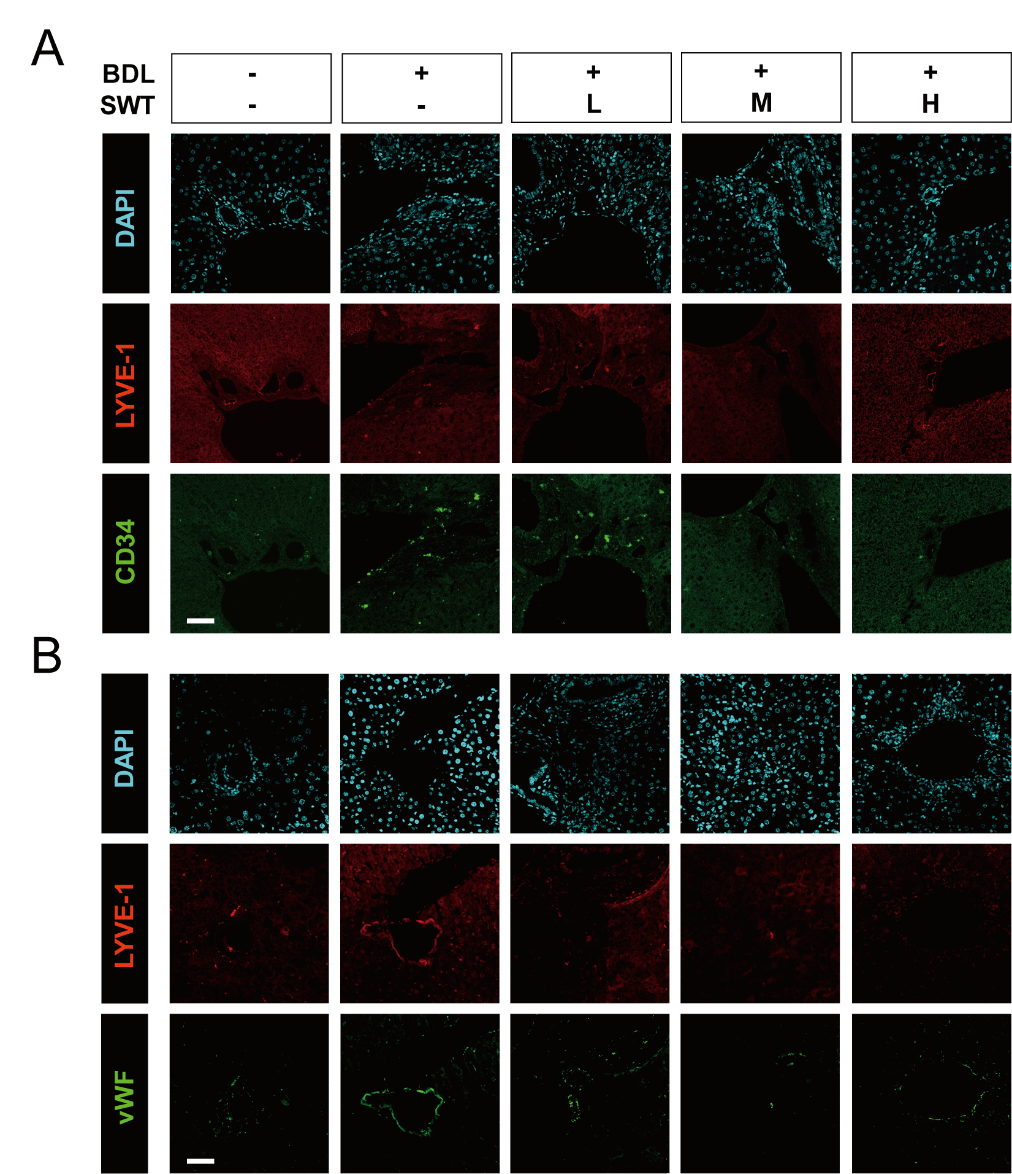


**Fig. S1. SWT regulates LSEC angiogenesis *in vivo*.** (**A, B**) Immunofluorescence co-staining of CD34 & LYVE-1 (**A**) and vWF & LYVE-1 (**B**) in mouse liver (scale bar = 50 μm).


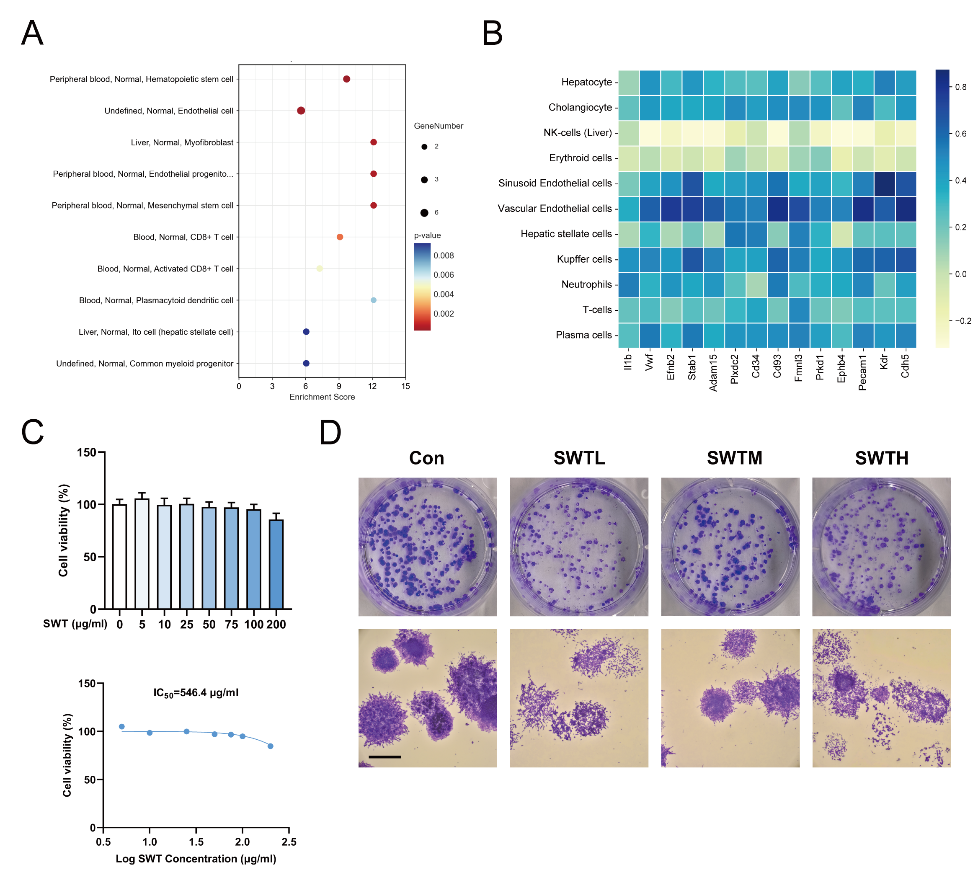


**Fig. S2. SWT targets LSECs to regulate liver fibrosis.** (**A**) The enrichment of different cell marker genes. (**B**) Expression of angiogenesis-related genes in different liver cells. (**C**) The impact of SWT on the cell viability of LSECs. (**D**) The impact of SWT on the proliferation of LSECs (scale bar = 200 μm).


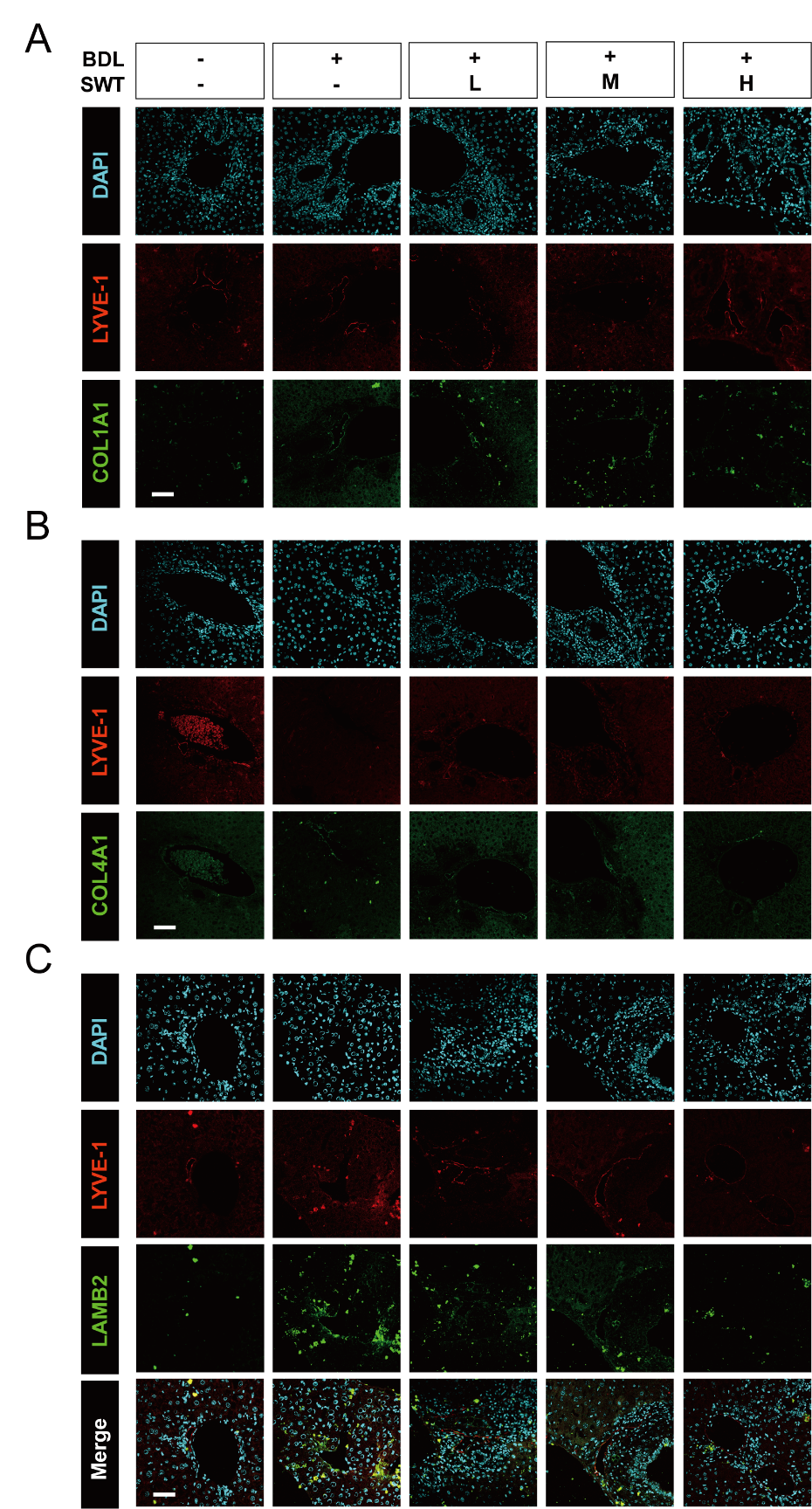


**Fig. S3. SWT inhibits LSEC basement membrane formation *in vivo*.** (**A-C**) Immunofluorescence co-staining of COL1A1 & LYVE-1 (**A**), COL4A1 & LYVE-1 (**B**) and LAMB2 & LYVE-1(**C**) in mouse liver (scale bar = 50 μm).


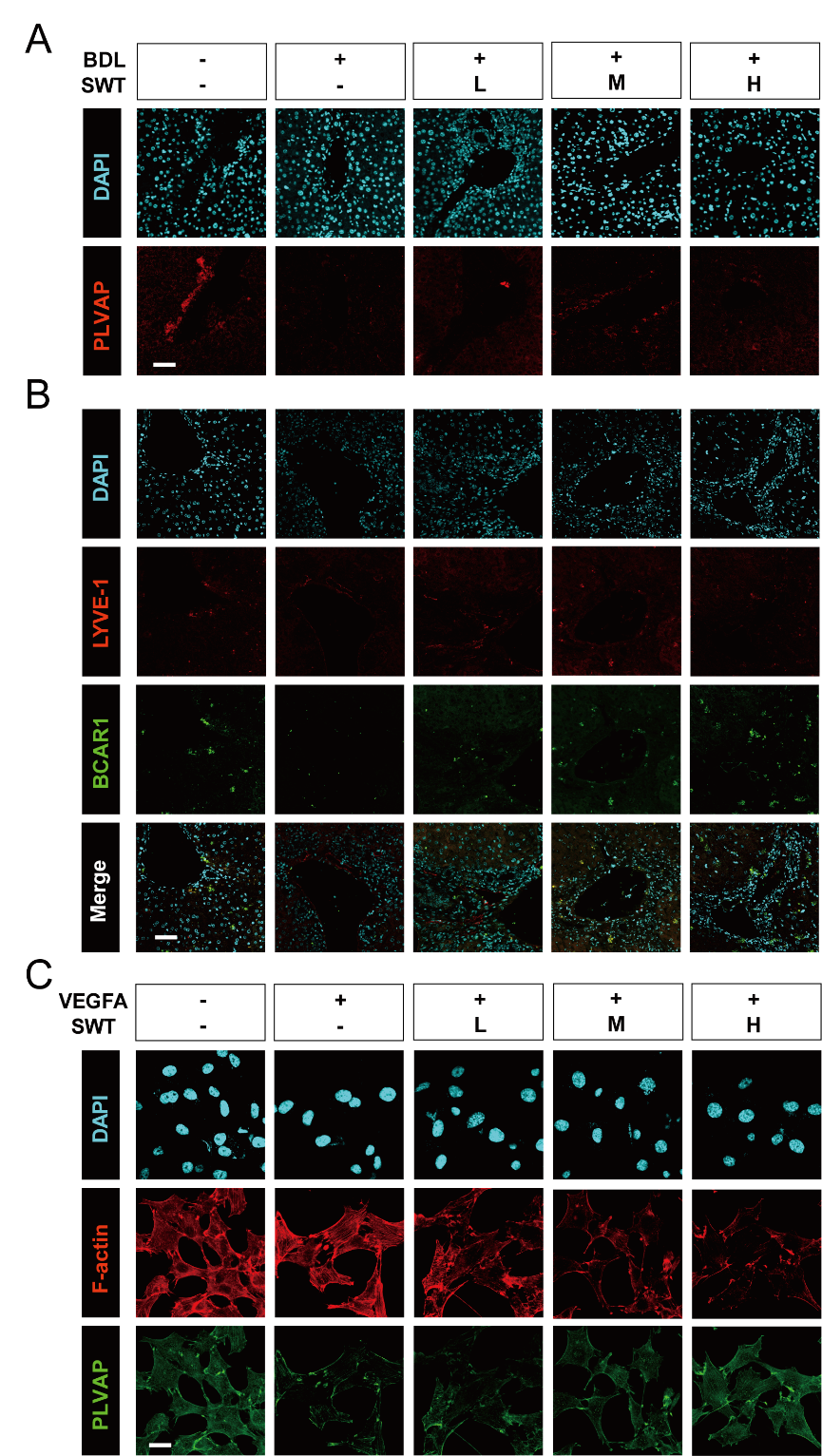


**Fig. S4. SWT restores LSEC fenestration *in vivo* and *in vitro*.** (**A**) Immunofluorescence staining of PLVAP. (**B**) Immunofluorescence co-staining of BCAR1 & LYVE-1 in mouse liver (scale bar = 50 μm). (**C**) Immunofluorescence co-staining of PLVAP & F-actin in rat LSECs (scale bar = 20 μm).


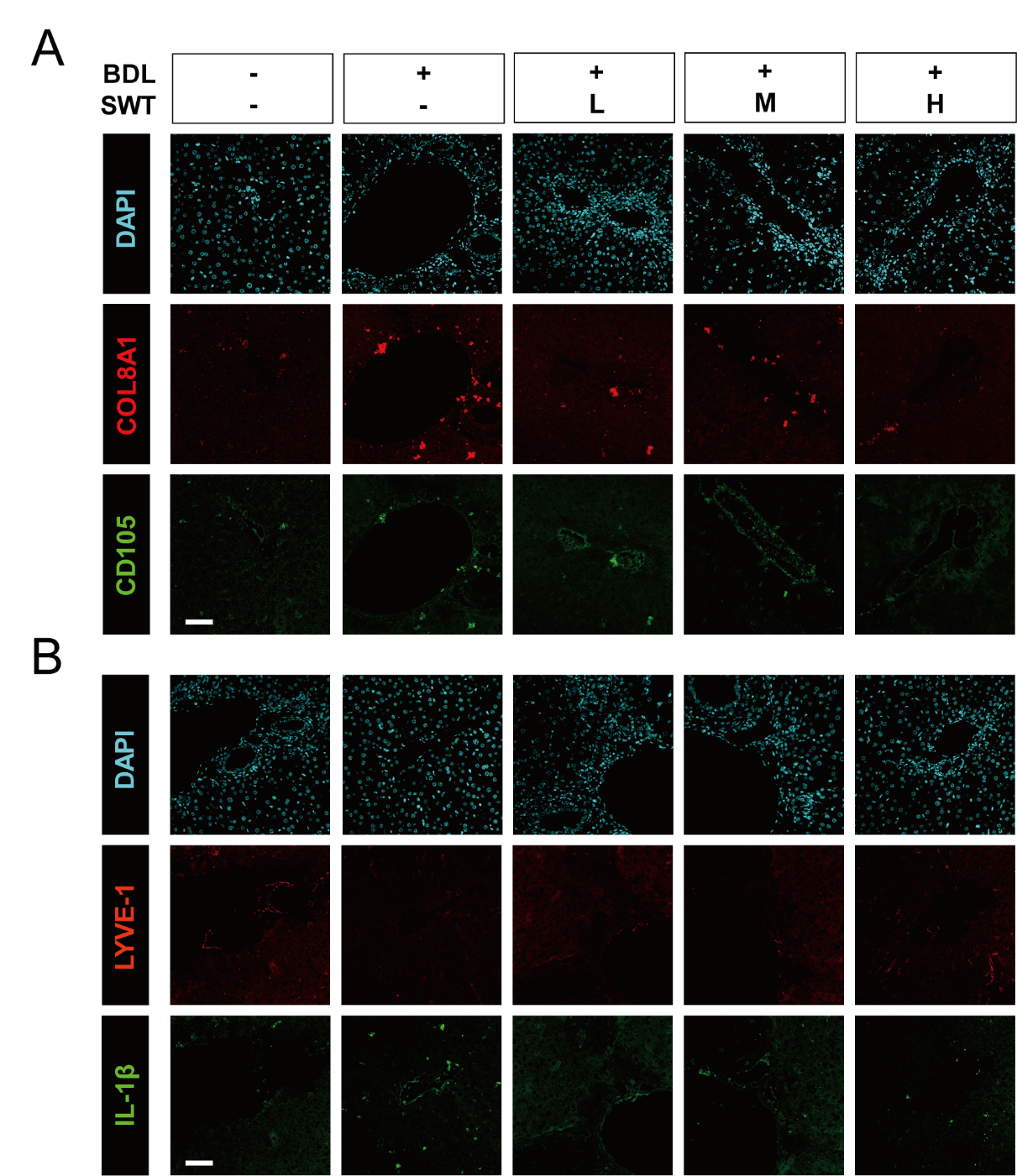


**Fig. S5. Regulation of COL8A1 and IL-1β by SWT *in vivo*.** (**A, B**) Immunofluorescence co-staining of COL8A1 & CD105 (**A**) and IL-1β & LYVE-1 (**B**) in mouse liver (scale bar = 50 μm).


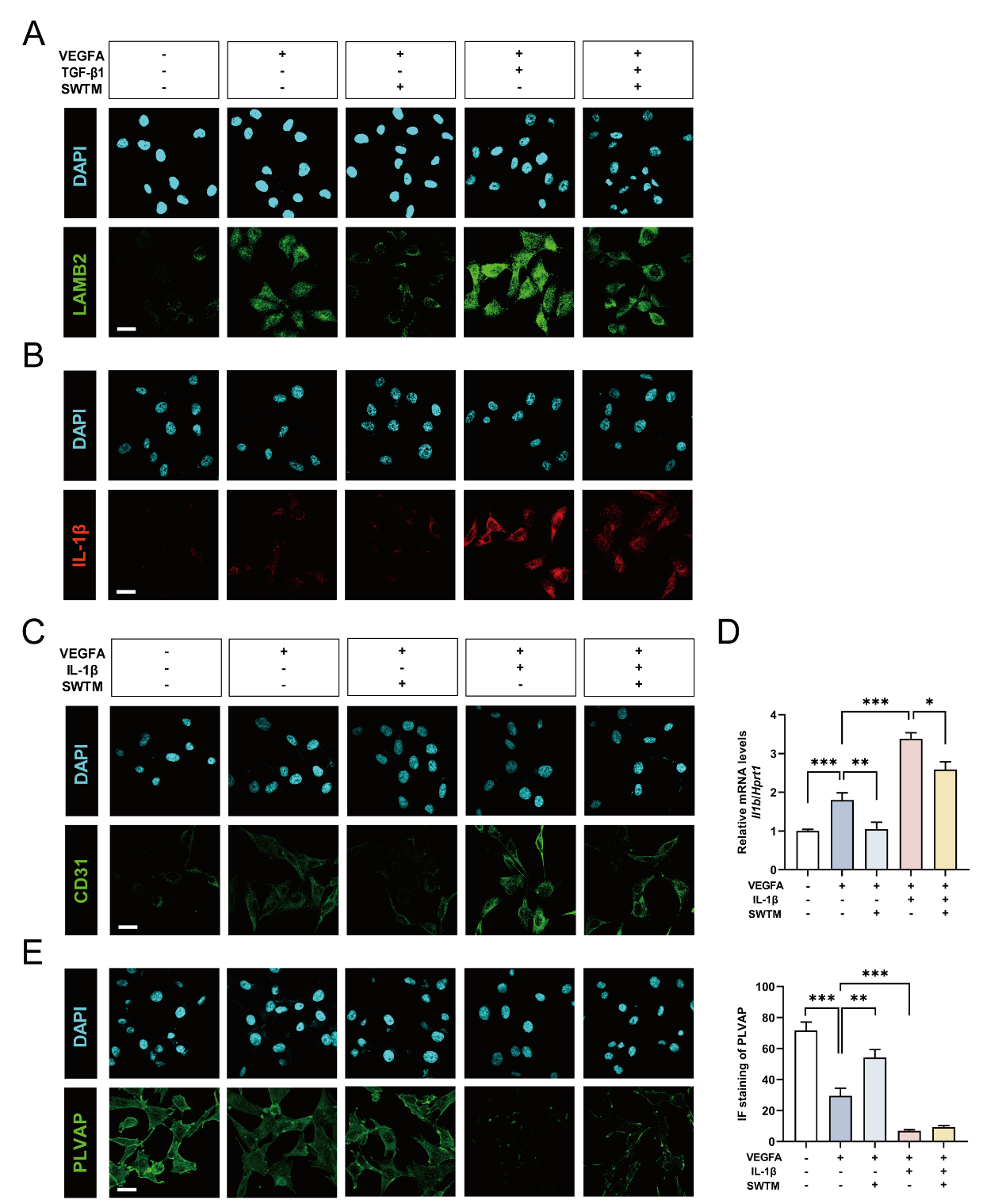


**Fig. S6. SWT modulates COL8A1/IL-1B/OLR1 pathway in LSECs.** (**A-C**) Immunofluorescence staining of LAMB2 (**A**) IL-1β (**B**) and CD31 (**C**) in rat LSECs (scale bar = 20 μm). (**D**) The qPCR results of relative mRNA levels of *Il1b* in rat LSECs compared to *Hprt1*. (**E**) Immunofluorescence staining of PLVAP (scale bar = 20 μm) and the statistical result. Data are shown as mean ± SEM, n = 6 (One-way ANOVA with Tukey’s post-hoc tests). **P* < 0.05, ***P* < 0.01, ****P* < 0.001 as compared with another group.


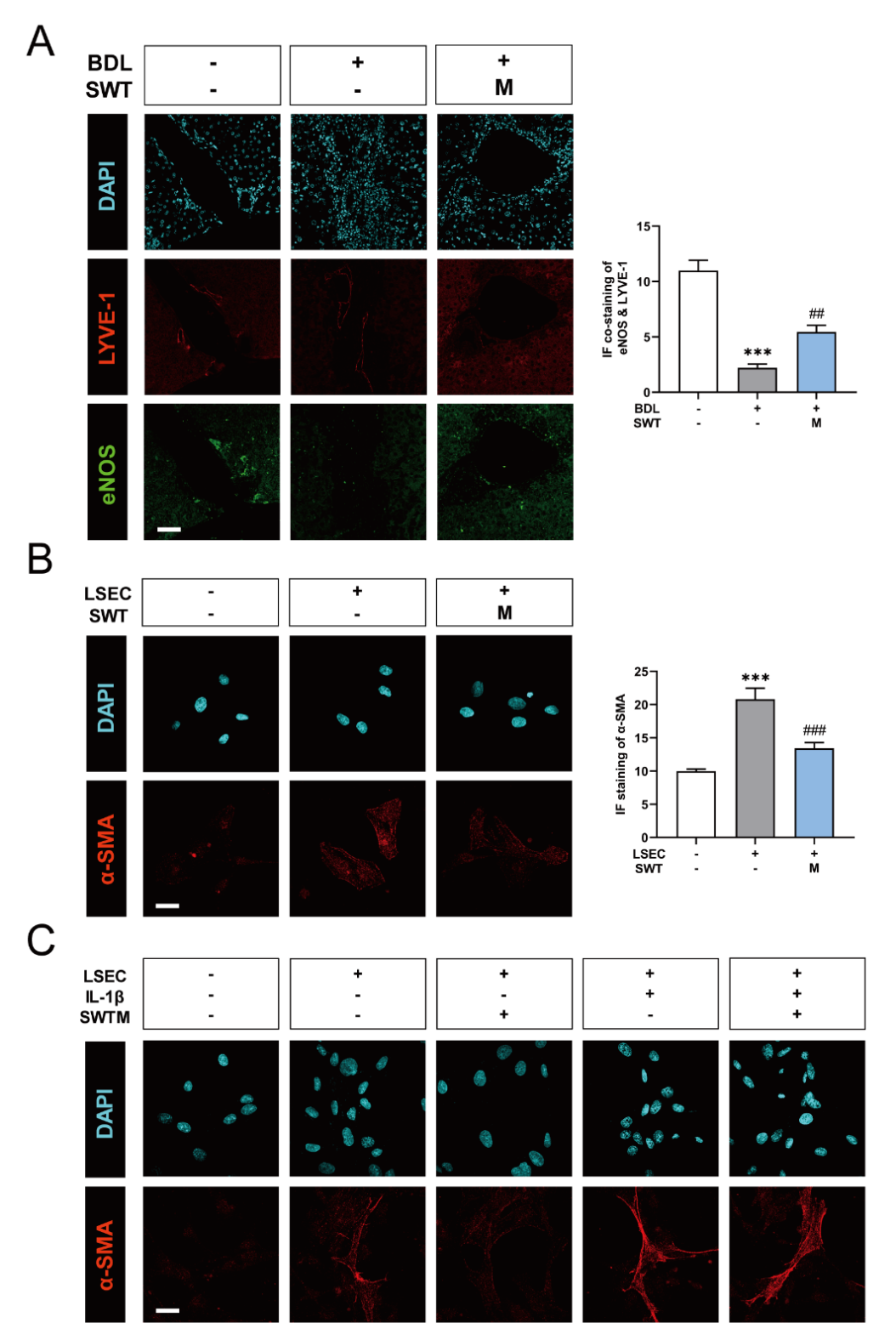


**Fig. S7. SWT regulates communication between LSEC and HSC.** (**A**) Immunofluorescence staining of eNOS & LYVE-1 in mouse liver (scale bar = 50 μm). (**B, C**) Immunofluorescence staining of α-SMA in rat HSC-T6 (scale bar = 20 μm). Data are shown as mean ± SEM, n = 6 (One-way ANOVA with Tukey’s post-hoc tests). ****P* < 0.001 as compared with the first group; ^##^*P* < 0.01, ^###^*P* < 0.001 as compared with the second group.

| **3. Supplementary table**  Table S1. **Antibody information** | | | |
| --- | --- | --- | --- |
| Antibodies | Source | Identifier | Dilution |
| Fibronectin Polyclonal Antibody | Proteintech | 15613-1-AP | 1:200 |
| EDN1 Polyclonal Antibody | Proteintech | 12191-1-AP | 1:1000 |
| β-Actin Monoclonal Antibody | Proteintech | 66009-1-Ig | 1:5000 |
| CD31 Monoclonal Antibody | Proteintech | 66065-2-Ig | 1:400 |
| CD34 Monoclonal Antibody | Proteintech | 60180-1-Ig | 1:300 |
| COL1A1 Monoclonal Antibody | Proteintech | 67288-1-Ig | 1:300 |
| COL8A1 Polyclonal Antibody | Proteintech | 17251-1-A | 1:150 |
| VEGF Monoclonal Antibody | Santa Cruz | sc-57496 | 1:500 |
| VEGFR2 Monoclonal Antibody | Santa Cruz | sc-6251 | 1:500 |
| vWF Monoclonal Antibody | Santa Cruz | sc-365712 | 1:300 |
| COL4A1 Monoclonal Antibody | Santa Cruz | sc-517572 | 1:50 |
| LAMB2 Monoclonal Antibody | Santa Cruz | sc-59980 | 1:300 |
| Rac Monoclonal Antibody | Santa Cruz | sc-514583 | 1:1000 |
| RhoA Monoclonal Antibody | Santa Cruz | sc-418 | 1:1000 |
| IL-1β Monoclonal Antibody | Santa Cruz | sc-52012 | 1:300 |
| p130 Cas Monoclonal Antibody | Santa Cruz | sc-20029 | 1:300 |
| CD105 Monoclonal Antibody | Santa Cruz | sc-18838 | 1:50 |
| eNOS Monoclonal Antibody | Santa Cruz | sc-376751 | 1:50 |
| LYVE-1 Monoclonal Antibody | CST | #67538 | 1:400 |
| PLVAP Monoclonal Antibody | CST | #38238 | 1:300 |
| α-SMA Monoclonal Antibody | CST | #19245 | 1:400 |
| Anti-rabbit IgG secondary antibody | CST | #8889 | 1:1000 |
| Goat anti-mouse IgG secondary antibody | Thermo Fisher | A-32723 | 1:1000 |
| Goat anti-rabbit IgG secondary antibody | Thermo Fisher | A-11008 | 1:1000 |

Table S2. **The components of SWT**

| Metabolites | Formula | m/z | T m/z | Rt (min) | Source |
| --- | --- | --- | --- | --- | --- |
| Rehmannioside D | C27H42O20 | 731.227 | 731.225 | 1.97 | RRP |
| 4-O-galloylalbiflorin | C30H32O15 | 633.181 | 633.181 | 5.07 | RPA |
| Levistolide A | C24H28O4 | 381.206 | 381.206 | 11.37 | RAS |
| Ligustilide | C12H14O2 | 191.107 | 191.107 | 10.17 | CR |
| Isoferulic acid | C10H10O4 | 195.065 | 195.065 | 5.07 | CR |
| Albiflorin | C23H28O11 | 481.17 | 481.17 | 4.56 | RPA |
| 3-Feruloylquinic acid | C17H20O9 | 367.103 | 367.103 | 4.6 | CR |
| Cnidilide | C12H18O2 | 195.138 | 195.138 | 10.11 | CR |
| Jionoside A1 | C36H48O20 | 799.268 | 799.267 | 4.52 | RRP |
| Lactiflorin | C23H26O10 | 507.151 | 507.151 | 5.51 | RPA |
| Verbascoside | C29H36O15 | 647.195 | 647.195 | 4.8 | RRP |
| Isomartynoside | C31H40O15 | 651.23 | 651.229 | 5.76 | RRP |
| Oxypaeoniflorin | C23H28O12 | 519.148 | 519.147 | 4.09 | RPA |
| 5-Hydroxyferulic acid | C10H10O5 | 193.05 | 193.05 | 4.75 | CR |
| 6'-O-galloylalbiflorin | C30H32O15 | 631.167 | 631.167 | 4.94 | RPA |
| Acetylcatalpol | C18H26O10 | 425.142 | 425.142 | 4.36 | RRP |
| Ajugol | C15H24O9 | 347.135 | 347.135 | 3.75 | RRP |
| Angelol B | C20H24O7 | 357.134 | 357.134 | 7.31 | RAS |
| Ethyl ferulic acid | C12H14O4 | 443.171 | 443.171 | 7.86 | CR |
| Angelol A | C20H24O7 | 399.142 | 399.141 | 7.64 | RAS |
| 3-O-Methylgallic acid | C8H8O5 | 183.029 | 183.03 | 3.8 | RPA |
| 5-Hydroxymethylfurfural | C6H6O3 | 127.039 | 127.039 | 2.53 | RRP |
| Isofraxidin | C11H10O5 | 221.045 | 221.046 | 5.18 | RAS |
| Mudanpiosidec | C30H32O13 | 599.178 | 599.177 | 5.55 | RPA |
| Catechin | C15H14O6 | 289.072 | 289.072 | 4.16 | RPA |
| Echinacoside | C35H46O20 | 785.252 | 785.251 | 4.33 | RRP |
| Ethyl Vanillate | C10H12O4 | 391.14 | 391.14 | 6.97 | CR |
| 3-Butylidenephthalide | C12H12O2 | 171.081 | 171.08 | 10.24 | CR |
| Butylphthalide | C12H14O2 | 191.107 | 191.107 | 9.56 | CR |
| Paeonol | C9H10O3 | 149.06 | 149.06 | 7.89 | RPA |

T = Theoretical, Rt = Retention time, RRP = Rehmanniae Radix Praeparata, RRA = Radix Paeoniae Alba, RAS = Radix Angelicae Sinensis, CR = Chuanxiong Rhizoma
